# Dynamic performance enhancement of adjustable blade pitch angle for wind generation system applications based on artificial neural network control techniques

**DOI:** 10.1038/s41598-026-53411-9

**Published:** 2026-05-26

**Authors:** Asmaa G. Ameen, Shuaiby Mohamed, Gamal T. Abdel-Jaber, I. Hamdan

**Affiliations:** 1 Mechanical Engineering Department, Faculty of Engineering, Qena University, Qena, 83523 Egypt; 2 Artificial Intelligence Department, Faculty of Computers and Artificial Intelligence, Hurghada University, Hurghada, 1950003 Egypt; 3 Mechatronics Engineering Department, Faculty of Engineering, Assiut University, Assiut, 71515 Egypt; 4 Department of Mechanical Engineering, Hanbat National University, Daejeon, 34158 South Korea; 5 New Assiut Technological University, Assiut, 71684 Egypt; 6 Department of Electrical Engineering, College of Engineering and Information Technology, Buraydah Private Colleges, Buraydah, 51418 Kingdom of Saudi Arabia; 7 Department of Electrical Engineering, Faculty of Engineering, Qena University, Qena, 83523 Egypt

**Keywords:** Wind speed variations, Blade pitch angle, PID, FPID, NN control, Energy science and technology, Engineering

## Abstract

The increasing reliance on the renewable energy, particularly wind power, introduces significant challenges for modern power systems and can compromise system stability. This study proposes an improved pitch-angle control strategy for a 1.5 MW large-scale Wind Energy Conversion System (WECS) based on a Doubly-Fed Induction Generator (DFIG). To address the limitations of conventional controllers, which struggle with system nonlinearity and the requirement for highly accurate mathematical models, this study examined Proportional-Integral-Derivative (PID) and Fractional PID (FPID) strategies. These were integrated with Neural Network (NN) architectures, specifically Multilayer Feedforward (MLFFNN), Cascade Forward (CFNN), and Elman NN, to improve control performance. The results, using MATLAB/Simulink, show that the MLFFNN architecture provides superior performance. With a minimum Mean Square Error of 0.0027024 and a power performance efficiency reaching a 98.9% under step, ramp, and random wind speed variations, the proposed NN controller consistently outperforms both PID and FPID systems, offering a robust solution for large-scale wind energy applications.

## Introduction

**Fig. 1:**
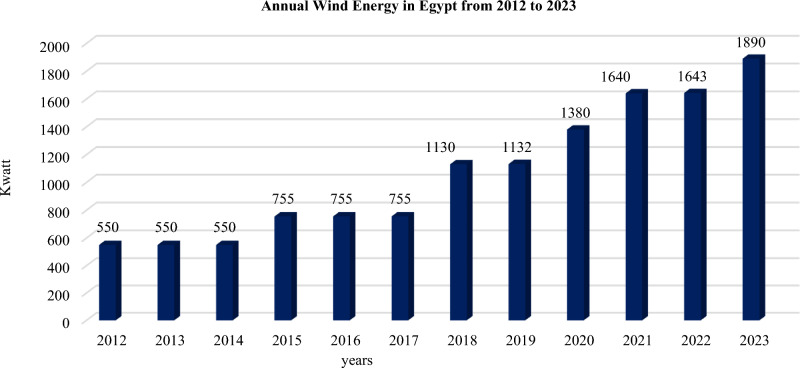
Egypt’s yearly wind energy from 2012 to 2023.

Green energy has become more popular because of the growing demand for alternative fuels, due to the devastation they cause to the environment and the ongoing increase in the world’s energy consumption. Wind energy is becoming increasingly important since it is clean, plentiful, and accessible^1^. Numerous Renewable Energy Sources (RESs) are regularly integrated into the power grid by energy regulators and researchers to mitigate risks associated with Traditional Energy Sources (TESs)^2^. Evidently, one of the main objectives of a Wind Energy Conversion System (WECS) is to provide the electrical grid with a steady, robust, and regulated power supply that is not impacted by the defects and outages^3^. To produce electricity, different RESs depend on atmospheric conditions such as wind speed, air temperature, atmospheric pressure, and humidity that directly affect power generation. Such circumstances are also essential for the grid operator’s guidance, which includes selecting wind turbine substations, enhancing the ingress of electrical power into the grid, and anticipating future energy demands and issues^4,5^. As wind energy becomes a major contributor to global electricity production, its integration into the power grid remains a key focus of ongoing research^6^. Estimates suggest that the global wind energy potential could reach approximately 1,011 Gigawatts (GW)^7^. Wind power plays a vital role in helping the electrical sector meet its energy and climate change commitments^8^. In Egypt, the installed wind energy capacity grew from 550 MW in 2012 to 1,890 MW by 2023, as illustrated in Fig. 1.[9]

There are two types of Wind Turbines (WTs), which are fixed-speed and variable-speed. Compared to fixed-speed turbines, Variable-Speed Wind Turbines (VSWTs) can capture more energy^10^. Many WT manufacturers are developing new megawatt-scale turbines with variable-speed operation, using pitch control and incorporating either permanent magnet synchronous generators (PMSGs) or doubly fed induction generators (DFIGs)^11^. The superior control capabilities of DFIGs have contributed to their widespread adoption in wind power plants^12,13^. WECS aims to extract the maximum possible power to ensure efficient operation. To achieve this goal, the wind-speed-dependent tip speed ratio (TSR) of the wind turbine (WT) is adjusted to its optimal value^14^. To maintain power output and maximize generator efficiency under varying wind conditions, the pitch angle must be properly regulated^15^. The rotor must operate at its rated speed to achieve maximum power transmission^16^. When wind speed exceeds the rated level, the pitch angle is increased to maintain the rotor speed at its rated value and maximize power output. Additionally, the controller must limit the rotor’s rotational speed to prevent damage during the highest wind conditions^17^. Therefore, pitch angle control has been proposed as a method for stabilising power output. It is commonly used to regulate both the rotor speed and the power output of wind turbines^18^.

As a result, adopting advanced control strategies for WECS is essential. Due to their simple construction and design, conventional Proportional-Integral (PI) controllers have a minimal impact on Digital Signal Processing (DSP). However, their poor dynamic response, characterised by overshoot, difficulty in tuning, and inability to handle unknown external disturbances, reduces both dynamic performance and control efficiency^19,20^. Sliding Mode Control (SMC) is one of the most effective nonlinear controllers under certain assumptions, offering excellent performance, fast dynamic response, and robustness^21^. In^22^, Integral Sliding Mode Control (ISMC) was developed using an integral sliding surface to address steady-state error and enhance the performance of the sliding surface issues. A multi-objective genetic algorithm was employed to improve the performance of DFIG in ^23,24^. In^25^, Artificial Neural Networks (ANNs) and genetic algorithms were proposed to enhance the reactive power regulation of Static Synchronous Compensator (STATCOM). The optimal STATCOM configuration integrated with Control Weight Function (CWF) parameters was identified in^26^ using the whale optimisation algorithm, genetic algorithm, and ANN.

To manage the pitch angle of the wind turbine system, an online training method was developed using a radial basis function (RBF) neural network to generate a self-adjusting controller^27^. In ^28^, an alternative approach applied fuzzy logic to establish a rule-based method for turbine selection. A neuro-fuzzy technique was developed by Asghar and Liu^29^ to determine the optimal rotor speed of a wind turbine. This method uses an Adaptive Neuro-Fuzzy Inference System (ANFIS) to estimate actual wind speed online, based on real-time values of the turbine’s tip speed ratio, rotor speed, and mechanical power. A variable pitch controller that combines a Proportional-Integral-Derivative (PID) controller with a Backpropagation (BP) neural network is discussed in^30^. This work suggests employing a Fractional Proportional-Integral-Derivative (FPID) and Neural Network (NN) controller to enhance WT optimal control performance by developing a classical PID. The effectiveness of the NN-based pitch-angle control in mitigating aerodynamic stresses and preserving mechanical power within its design limits was confirmed by comparison between FPID, NN, and traditional PID control strategies.

The main challenges of this study are controlling system nonlinearity, time delays, and the drawbacks of traditional control techniques, which depend on the accurate system modelling aims to enhance dynamic performance by successfully altering the blade pitch angle of wind turbines under varying wind speeds. ANN-based control strategies are employed to address this challenge, enhancing the instantaneous flexibility and efficiency of wind turbine operations.

The following points represent an outline of this study’s contributions:

- Proposed a pitch-angle adjustment control technique based on ANNs.
- Improved wind turbine dynamic performance under instances of variable wind speed.
- Enhanced reactivity, flexibility, and general efficiency of wind turbine operations.
- Assisted in developing wind energy control systems that are more complex.

The rest of this study is organised as follows: Section "Modelling of wind turbine" introduces the modelling of the wind turbine’s output power. The dynamic model of the DFIG, explaining its behaviour and key components is presented in Section "DFIG dynamic model". Section "Control Strategy" describes the control strategies employed to enhance wind turbine performance, including PID, FPID, and neural network approaches. Section "Simulation Results and Discussions" illustrates simulation results of applying PID, FPID, and NN controllers to the WECS. A comparison with previous research is provided in Section "Comparison with Previous Research". Finally, conclusions and future work are presented in Section "Conclusions and Future Work".

## Modelling of wind turbine

Using a DFIG, Variable-Speed Wind Turbines (VSWTs) convert kinetic energy into mechanical energy, then to electrical power. The physical definition of kinetic energy is provided as follows^31,32^:

1 $$KE=\frac{1}{2} m {V}^{2}$$

where $m$ is the mass of air ($Kg$) and $V$ is the wind speed (m/s). On the other hand, the rate of change of kinetic energy is defined as power ${P}_{m}$:

2 $${P}_{m}=\frac{1}{2}\frac{dm}{dt}{V}^{2}$$

3 $$\frac{dm}{dt}=\rho {A}_{s}V, {A}_{s}=\pi {r}^{2}$$

where $r$ is the rotor radius ($m$),${A}_{s}$ is the swept area of blades (${m}^{2}$) and ρ is the air density ($Kg/{m}^{3}$).

The relationship between air density, power coefficient, turbine-swept area, and captured energy is expressed as

4 $$P_{m} = \frac{1}{2}A_{s} \rho C_{p} \left( {\reflectbox{$\lambda$} ,\beta } \right)V^{3}$$

With;

5 $$\reflectbox{$\reflectbox{$\lambda$}$} = \frac{wR}{V}$$

where $${P}_{m}$$, $$\reflectbox{$\lambda$} ,\beta$$, $${C}_{p}$$ and ω are mechanical power, TSR, pitch angle, power coefficient and rotor shaft speed, respectively.

**Fig. 2:**
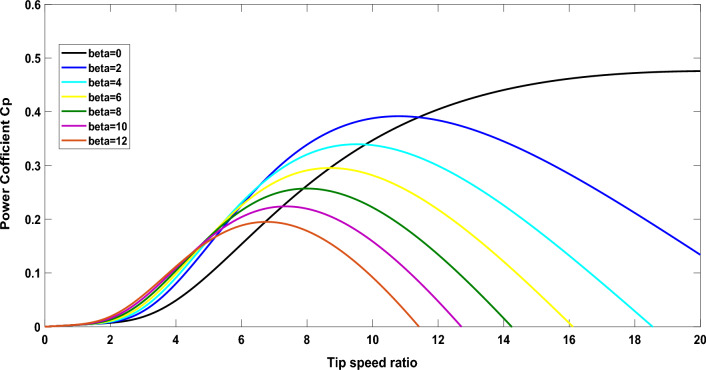
Modification of ${\mathrm{C}}_{\mathrm{p}}$ with ʎ for different values of β.

Entirely, the pitch-angle $$\left( \beta \right)$$ and TSR $$\left( \reflectbox{$\lambda$} \right)$$are nonlinear variables which provide an operating coefficient ($${C}_{p}$$) as shown in Fig. 2, which is indicated as follows:

6 $$C_{p} \left( {\reflectbox{$\lambda$} ,\beta } \right) = k_{1} \left( {\frac{{k_{2} }}{{\reflectbox{$\lambda$}_{i} }} - k_{3} \beta - k_{4} \beta^{{k_{5} }} - k_{6} } \right)e^{{\frac{{ - k_{7} }}{{\reflectbox{$\lambda$}_{i} }}}}$$

7 $$\reflectbox{$\lambda$}_{i} = \left[ {\frac{1}{{\left( {\reflectbox{$\lambda$} + k_{8} \beta } \right)}} - \frac{{k_{9} }}{{\left( {\beta^{3} + 1} \right)}}} \right]^{ - 1}$$

The parameters of the wind turbine model are denoted by the constants presented in Table 1^33^.

**Table 1:** The values of the kinetic coefficients.

Constant	Value
${{\boldsymbol{k}}}_{1}$	0.73
${{\boldsymbol{k}}}_{2}$	151
${{\boldsymbol{k}}}_{3}$	0.58
${{\boldsymbol{k}}}_{4}$	0.002
${{\boldsymbol{k}}}_{5}$	2.14
${{\boldsymbol{k}}}_{6}$	13.2
${{\boldsymbol{k}}}_{7}$	18.4
${{\boldsymbol{k}}}_{8}$	0.02
${{\boldsymbol{k}}}_{9}$	0.003

## DFIG dynamic model

**Fig. 3:**
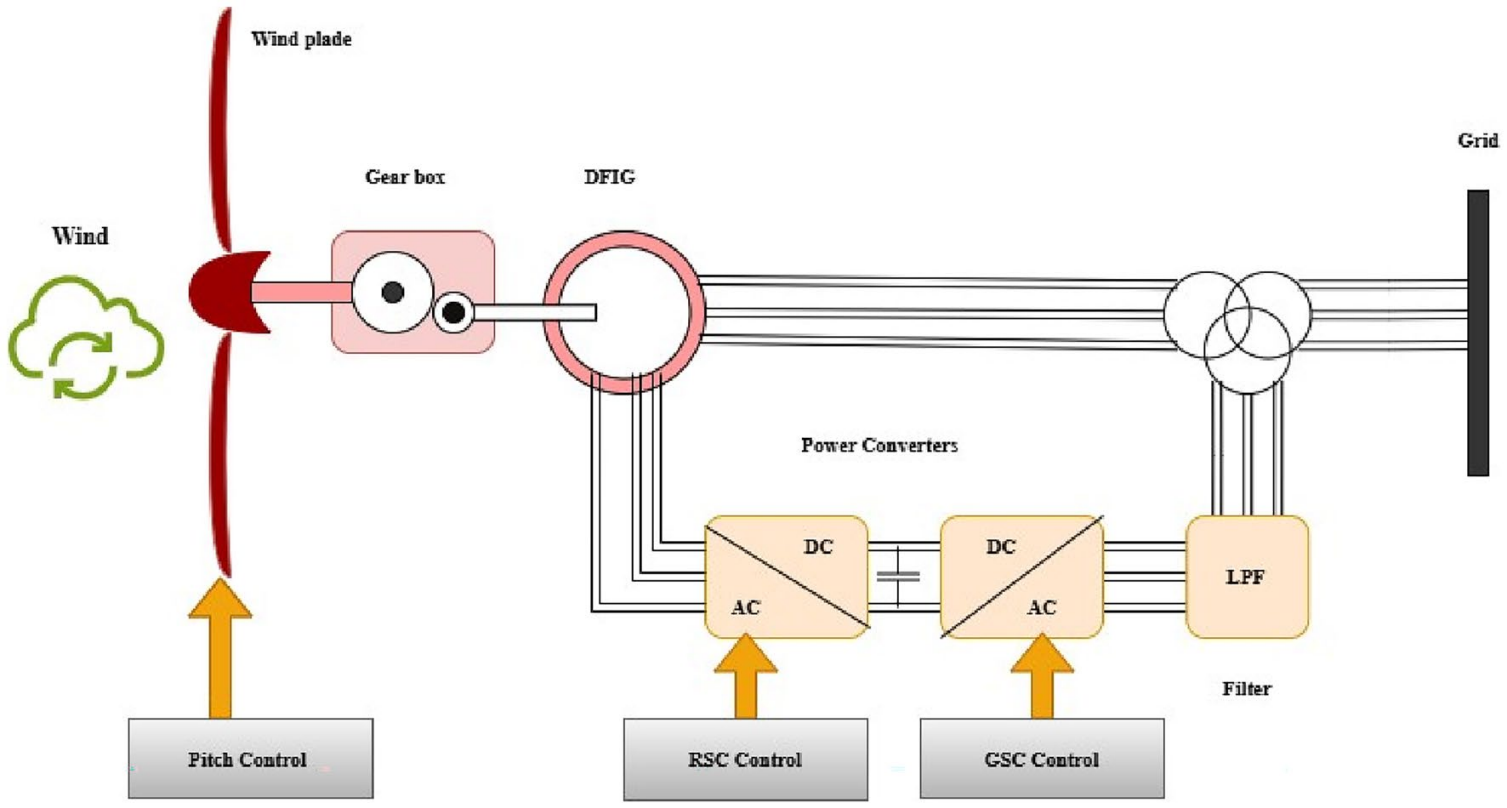
Block diagram of DFIG.

An interconnected grid block diagram of the DFIG is shown in Fig. 3.

The DFIG voltage formulas and the rotor and stator flux connections are as follows ^34,35^:

8 $${\Psi}_{r}={L}_{r}{i}_{r}+{L}_{m}{i}_{s}$$

9 $${\Psi}_{s}={L}_{s}{i}_{s}+{L}_{m}{i}_{r}$$

10 $${V}_{r}={R}_{r}{i}_{s}+\frac{d}{dt}{\Psi}_{r}-j \omega {\Psi}_{r}$$

11 $${V}_{s}={R}_{s}{i}_{s}+\frac{d}{dt}{\Psi}_{s}$$

where ${L}_{s},{L}_{r}$ and ${L}_{m}$ are stator, rotor, and magnetising inductances; $j$ is an arbitrary unit; and ${R}_{r}$ and ${R}_{s},$ are the rotor and stator resistances in the system.

Below is an expression for the stator’s output active and reactive power:

12 $${P}_{s}=\frac{3}{2}({V}_{ds}{i}_{ds}+{V}_{qs}{i}_{qs})$$

13 $${Q}_{s}=\frac{3}{2}({V}_{qs}{i}_{ds}-{V}_{ds}{i}_{qs})$$

where ${P}_{s},{Q}_{s}$ represents the stator’s active and reactive powers, respectively and ( ${V}_{ds}$,${V}_{qs}$,${i}_{ds}$,${i}_{qs}$) are the dynamic voltages and currents in arbitrary units $d-q$ reference frame.

**Fig. 4:**
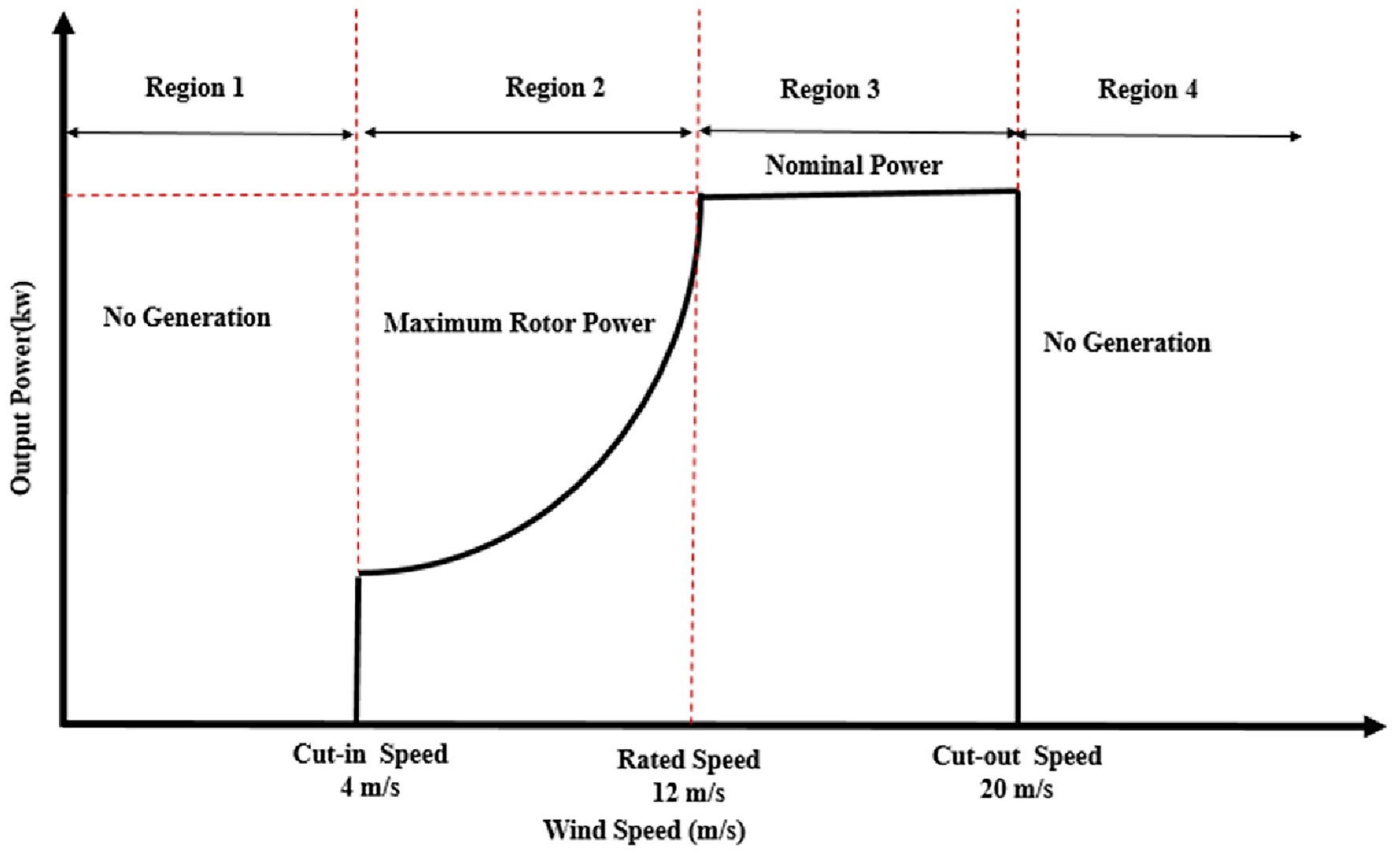
Working regions for a wind turbine.

Figure 4 depicts how the WT control approach was separated into four zones based on wind speed ^36^.

- Region 1: The region of standstill, the mechanical power generation is close to zero.
- Region 2: The WT operates to obtain the maximum amount of power during differences in wind speed in the MPPT zone.
- Region 3: Pitch control must be employed to restrict the rated output power of a wind turbine in the sloped working range.
- Region 4: To prevent damage at severe wind speeds, the WT need to be halted.

## Control Strategy

This study employs three control techniques: Proportional Integral Derivative (PID), Fractional Proportional Integral Derivative controller (FPID) and Neural Networks (NN).

### control technique

**Fig. 5:**
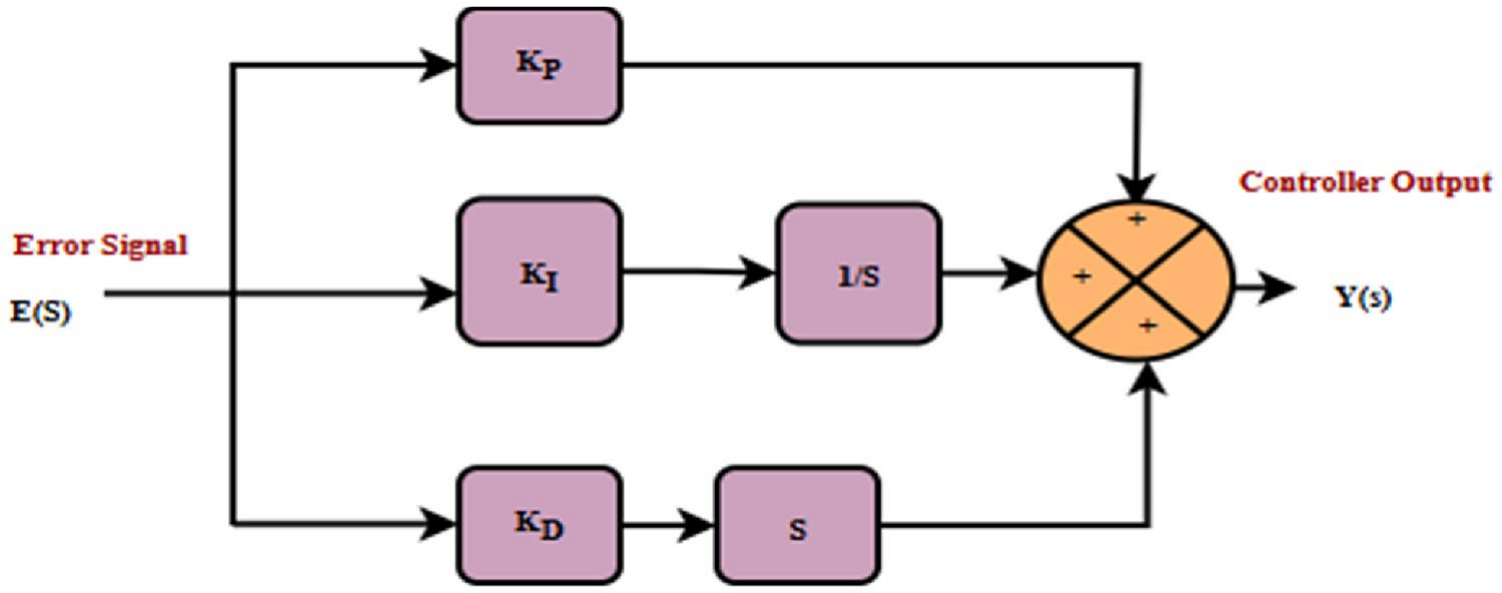
PID schematic model.

The PID controller has been recognised as the conventional control architecture in classical control theory. The schematic model of the control system using a general PID controller is shown in Fig. 5.

Equation 14 represents a mathematical expression of the linear relationship between the error $E(S)$, and the controller output $Y(S)$.

14 $$Y\left(S\right)={[K}_{P}+{K}_{I }\frac{1}{S}+{K}_{D} S] E(S)$$

where ${K}_{P }, {K}_{I }, \mathrm{a}\mathrm{n}\mathrm{d} {K}_{D}$ are the proportional, integral, and derivative gains, respectively.

**Fig. 6:**
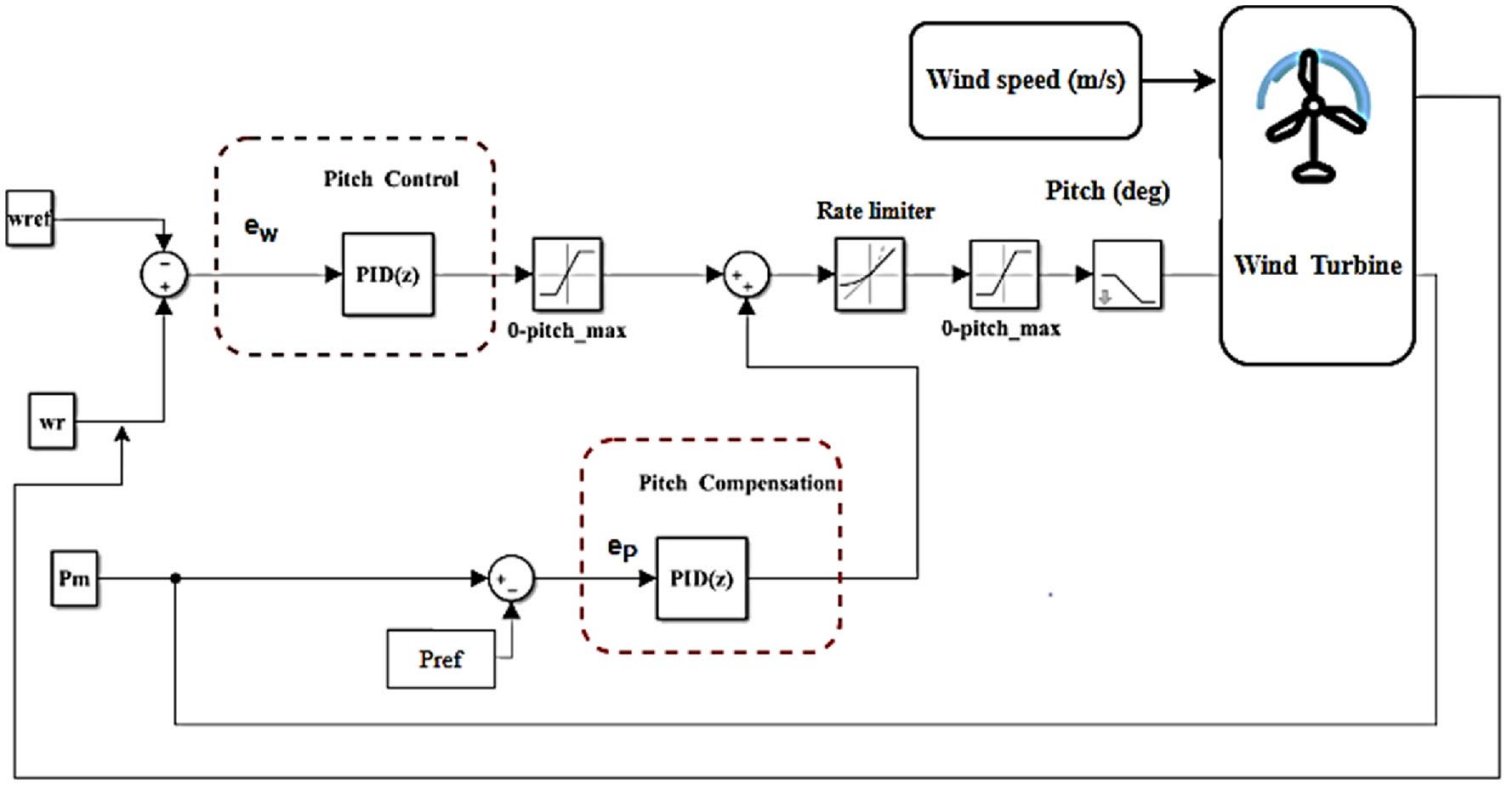
PID controller of pitch angle in wind turbine system.

As shown in Fig. 6, a PID controller was used to implement the pitch angle to the wind turbine system in MATLAB SIMULINK.

The rotor speed error ${e}_{w}(t)$ and power error ${e}_{p}(t)$ are implemented to control the output power to the rated value to accomplish the desired pitch angle. The performance of a traditional control depends entirely on its gains, as the output pitch angle of a PID controller is specified by ^37^. Table 2 presents the gains of the PID controller.

15 $$\beta ={K}_{P}\left({e}_{p}\left(t\right)+{e}_{w}\left(t\right)\right)+{K}_{I}\left({\int}_{0}^{t}{e}_{p}\left(t\right)+{\int}_{0}^{t}{e}_{w}\left(t\right)\right)+{K}_{D}\left(\frac{d{e}_{p}\left(t\right)}{dt}+\frac{d{e}_{w}\left(t\right)}{dt}\right)$$

16 $${e}_{p}\left(t\right)={P}_{m}-{P}_{ref}$$

where ${P}_{m}$ and ${P}_{ref}$ are measured output power and the reference value of output power, respectively

17 $${e}_{w}\left(t\right)={w}_{r}-{w}_{ref}$$

where ${w}_{r}$ and ${w}_{ref}$ are the measured rotor speed and the reference value of rotor speed.

**Table 2:** PID controller gains.

Gains		${{\boldsymbol{K}}}_{{\boldsymbol{P}}}$	${{\boldsymbol{K}}}_{{\boldsymbol{I}}}$	${{\boldsymbol{K}}}_{{\boldsymbol{D}}}$
Value		39	9	0.4

### FPID control technique

**Fig. 7:**
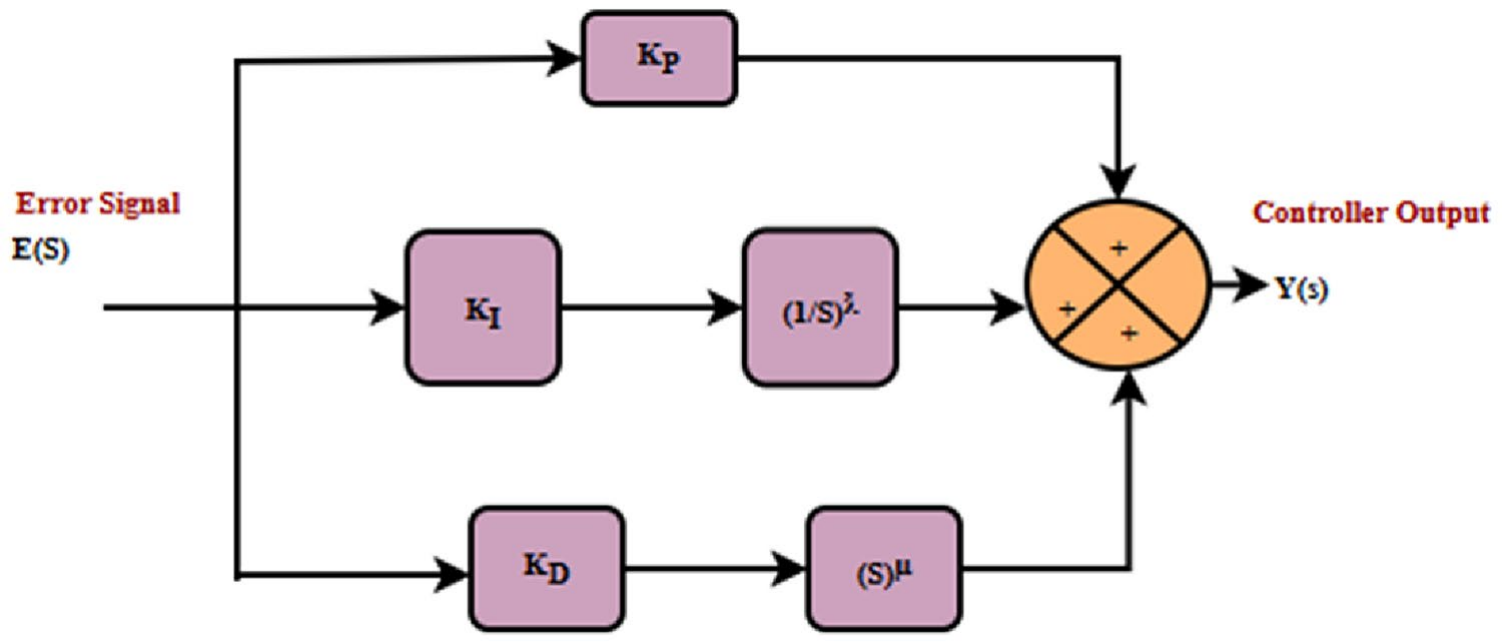
FPID schematic model.

Recently, an innovative modification of the traditional PID controller, defined as the FPID controller, has been widely studied. The magnitudes of the derivative and integrator, which are fractional, offer more flexibility than a PID controller, as shown in Fig. 7^38^. That can include the differentiator and integrator in the corresponding order of ƛ and µ. For the regulated system, the fractional orders ƛ and µ can be applied to accommodate requirements design or other relevant needs ^39^.

Equation 18 represents a mathematical expression of the linear relationship between the error $E(S)$, and the controller output $Y(S)$.

18 $$Y\left( S \right) = [K_{P} + K_{I } \left( \frac{1}{S} \right)^{\mathchar'26\mkern-10mu\lambda } + K_{D} s^{\mu } ] E\left( S \right)$$

where ${K}_{P}$ is the proportional gain,${K}_{I}$ is the integral gain, ${K}_{D}$ is the derivative gain and ƛ, µ are the fractional values.

**Fig. 8:**
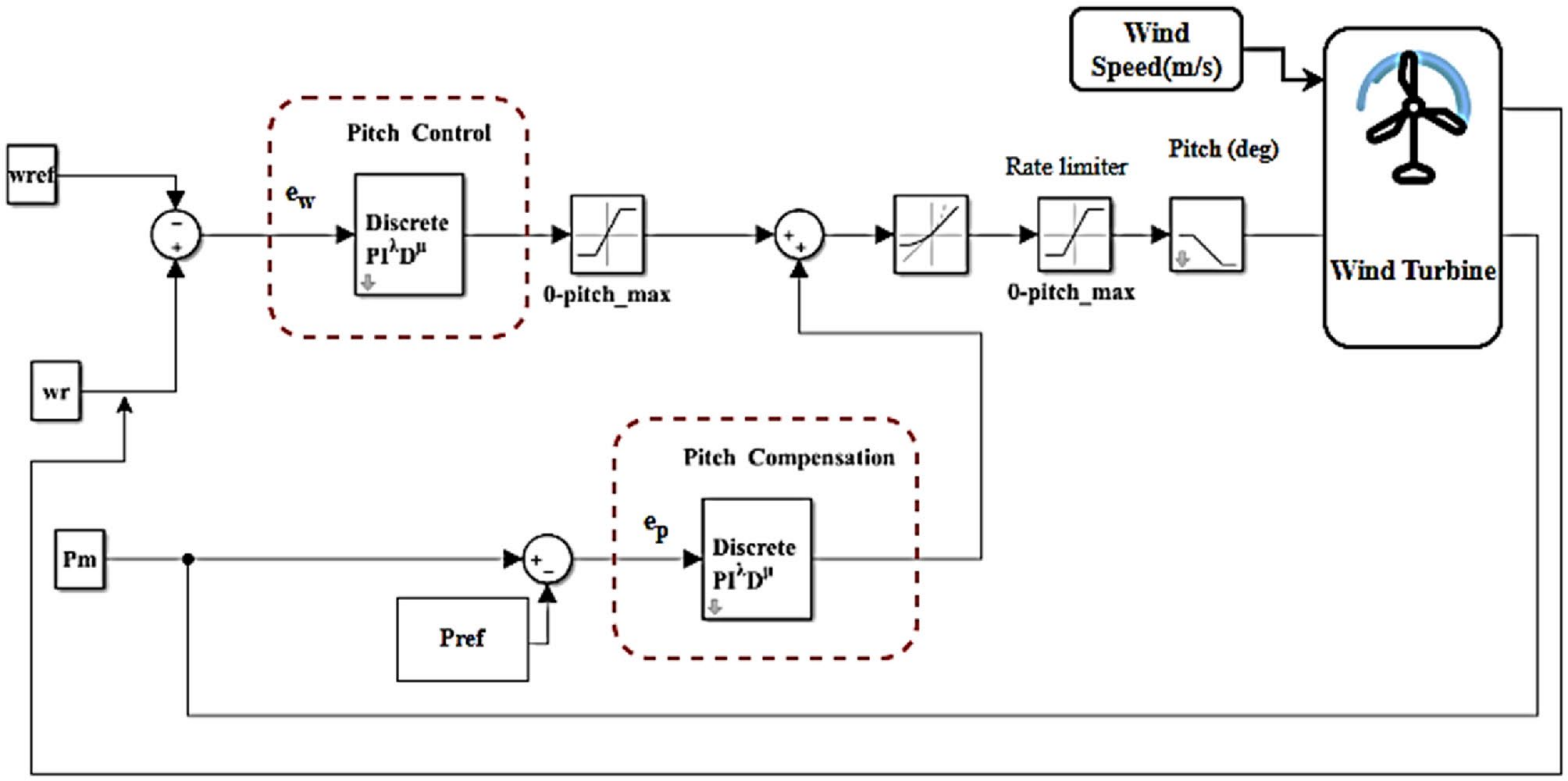
FPID controller of pitch angle in wind turbine system.

As shown in Fig. 8, an FPID controller was used to implement the pitch angle in the wind turbine system in MATLAB SIMULINK.

The FPID $(PI^{\mathchar'26\mkern-10mu\lambda } D^{\mu } )$ controller can be expressed mathematically using the differential equation that follows:

19 $$\beta = K_{P} (e_{p} (t) + e_{w} (t)) + K_{I} D_{t}^{{( - \mathchar'26\mkern-10mu\lambda )(e_{p} (t) + e_{w} (t))}} + K_{D} D_{t}^{\mu } (e_{p} (t) + e_{w} (t))$$

Table 3 presents the parameters of the FPID controller.

**Table 3:** FPID controller gains.

Gains	${{\boldsymbol{K}}}_{{\boldsymbol{P}}}$	${{\boldsymbol{K}}}_{{\boldsymbol{I}}}$	${{\boldsymbol{K}}}_{{\boldsymbol{D}}}$	ƛ	µ
Value	37	8	0.4	0.3	0.1

### NN control technique

NNs are data-handling and identification systems built up of a huge number of important and highly interconnected structural components that resemble the architecture of the actual sensory system ^40^. NNs are primitive electronic models that are based on the cognitive cortex’s neural architecture ^41^. Exceptional training algorithms that are designed based on criteria for learning are used to assist with learning in NNs to emulate biological systems’ learning processes ^42^.

#### MLFFNN technique

**Fig. 9:**
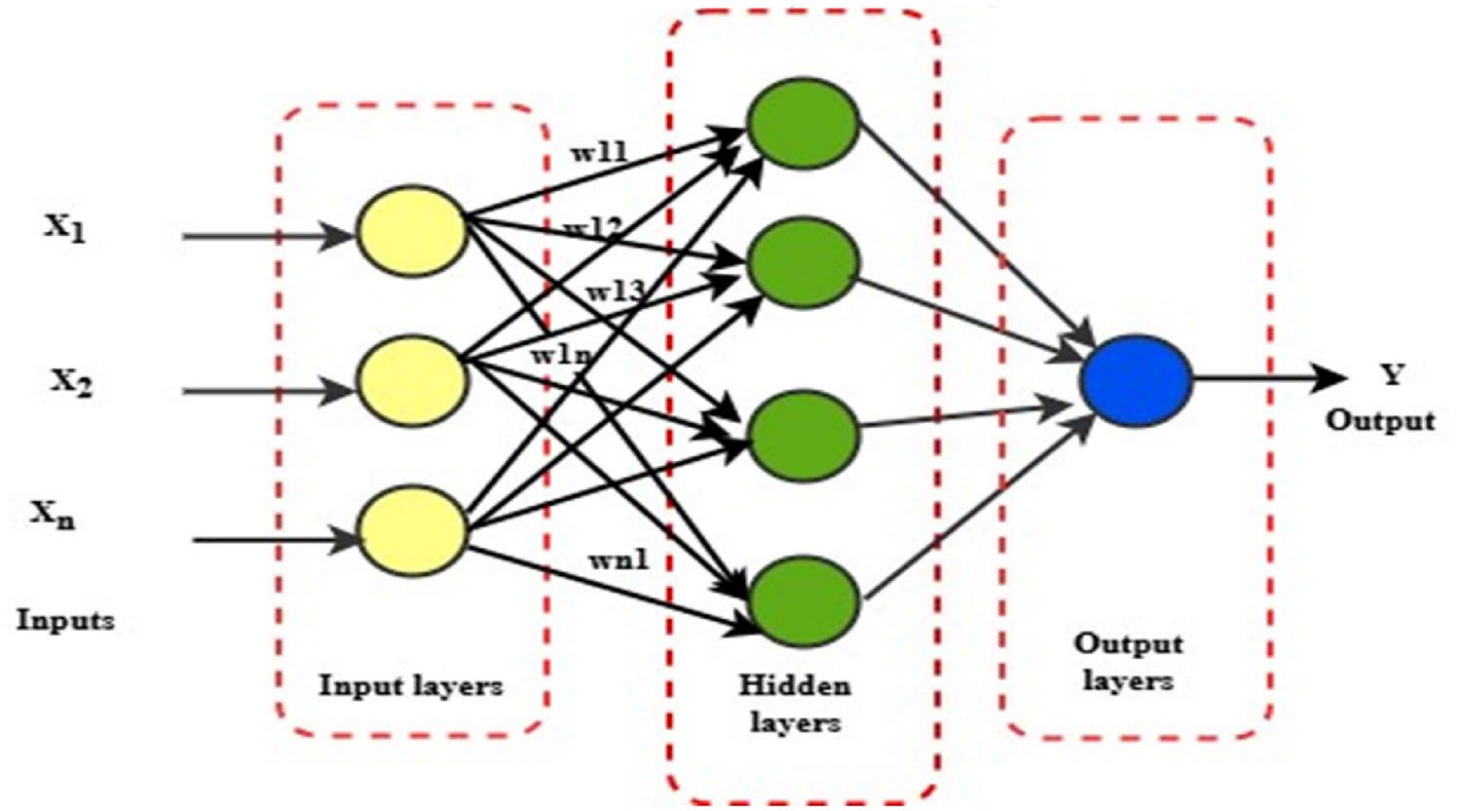
MLFFNN schematic diagram.

In this study, the most used type of feedforward network, the multilayer one, is taken into consideration. The MLFNN training procedure includes a gradual modification of the connection weights that create connections or pass information along neurons, which are primitive processing units. As demonstrated in Fig. 9, the MLFFNN architecture consisted of three layers: the input layer, the nonlinear hidden layer, and the output layer ^43,44^.

A single layer or multiple layers could make up the hidden layer. The hidden layer is employed to transmit the input signal from the input layer to the output layer.

Through the weight matrix W, the input was delivered to the neurons. The output can be obtained by ^45^:

20 $${y}_{j}={\Psi}_{j}\left({h}_{j}\right)={\Psi}_{j}{\sum}_{i=0}^{i=n}{X}_{i}{W}_{ji}$$

The NN’s output is symbolised by ${Y}_{j}$, the synaptic weights between the input and hidden neurons are expressed by ${W}_{ji}$, and the input signal, $X$, spans 1 to n inputs. The activation function for hidden layers is shown by Eq. (20).

21 $${\Psi}_{j}\left({h}_{j}\right)=\mathrm{t}\mathrm{a}\mathrm{n}\mathrm{h}({h}_{j})$$

The output power ${y}_{out}$ of the MLFFNN is given by:

22 $${y}_{out }={\Psi}_{k}\left(0\right)={\Psi}_{k}\left({\sum}_{j}^{n}{b}_{1j}{y}_{j}\right)=tanh.\left({\sum}_{j}^{n}{b}_{1j}{y}_{j}\right)$$

Where ${b}_{1j}$ is the weight between the hidden neurons $j$ and ${y}_{out}$ is the output power was generated by the MLFFNN.

23 $$error={y}_{desired}-{y}_{out}$$

**Fig. 10:**
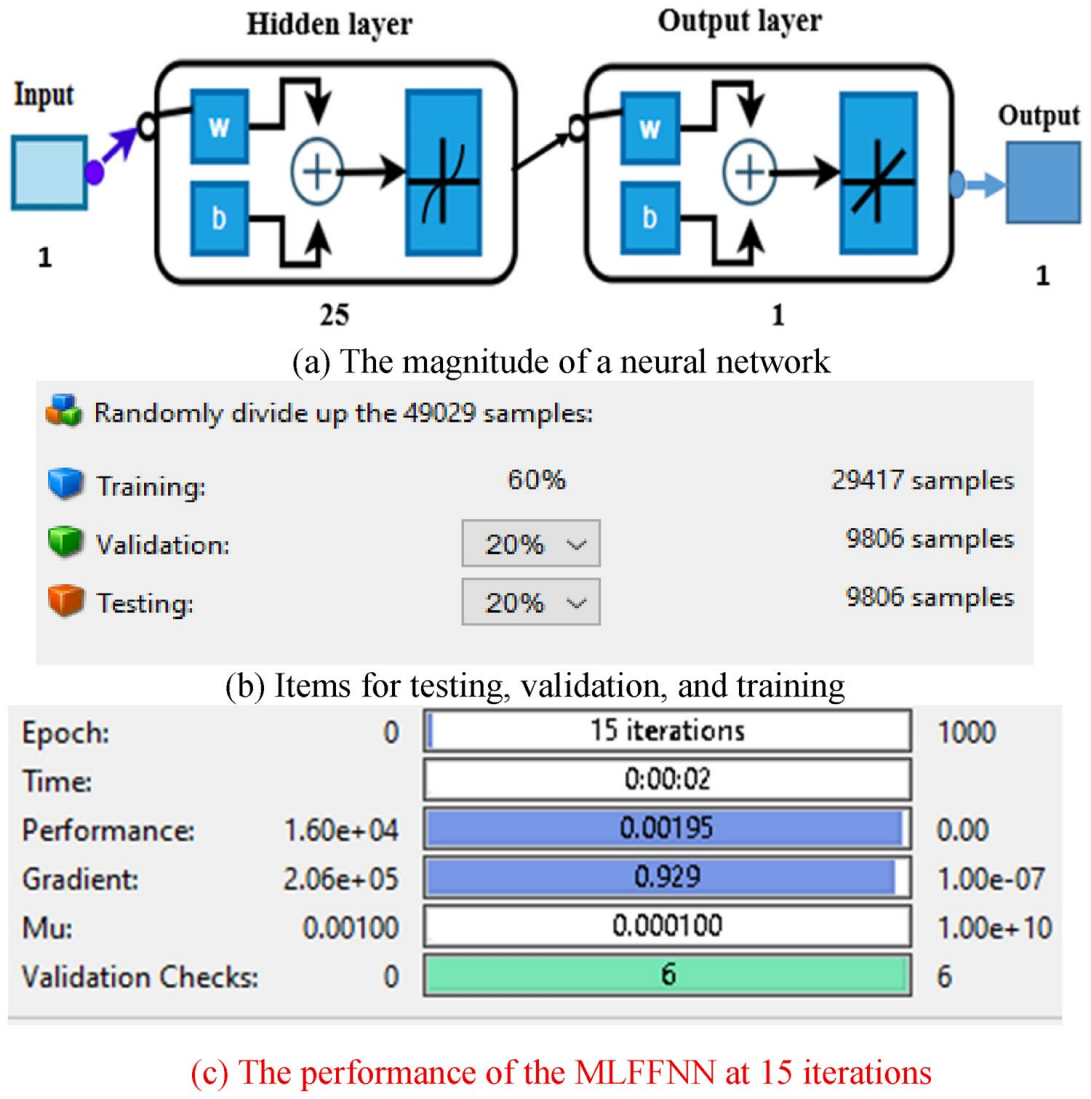
The NFTOOL Parameters of MLFFNN.

**Fig. 11:**
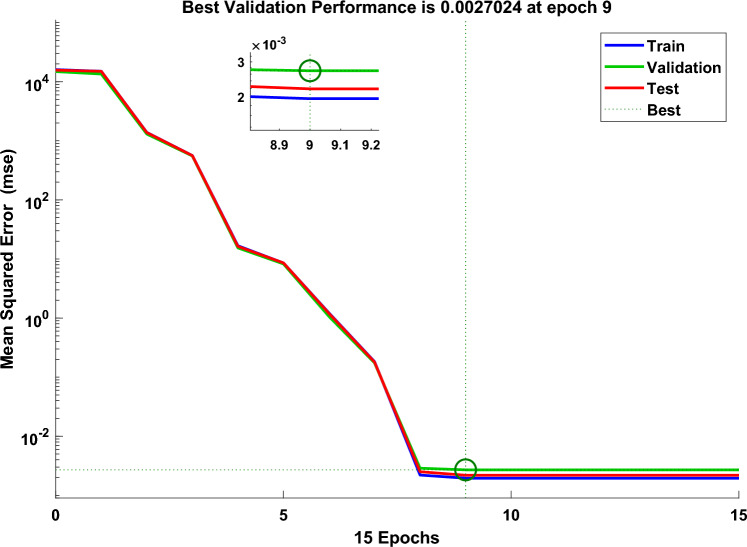
Integration of the studied system’s performance with MLFFNN.

The technique of Levenberg–Marquardt is implemented in this work to train the PC’s control parameter values. A combination of operations called training, validation, and testing makes up the neural fitting tool (NFTOOL). The program separates the input (error of rotor speed at pitch control and error of output power at pitch compensation) and target (pitch control parameters) into the following three groups: Training comprises 60% of the data. 20% is used for testing, while 20% is used for validation. The NFTOOL parameters are listed in Fig. 10, and Fig. 11 shows the results of the training process.

A Fig. 11 demonstrates the best validation efficiency of MLFFNN, the error decreases rapidly during the early epochs, indicating efficient learning. The best validation performance is achieved around epoch 8, after which the error stabilizes. The close alignment between training, validation, and test curves suggests good generalization and no significant overfitting.

#### CFNN technique

**Fig. 12:**
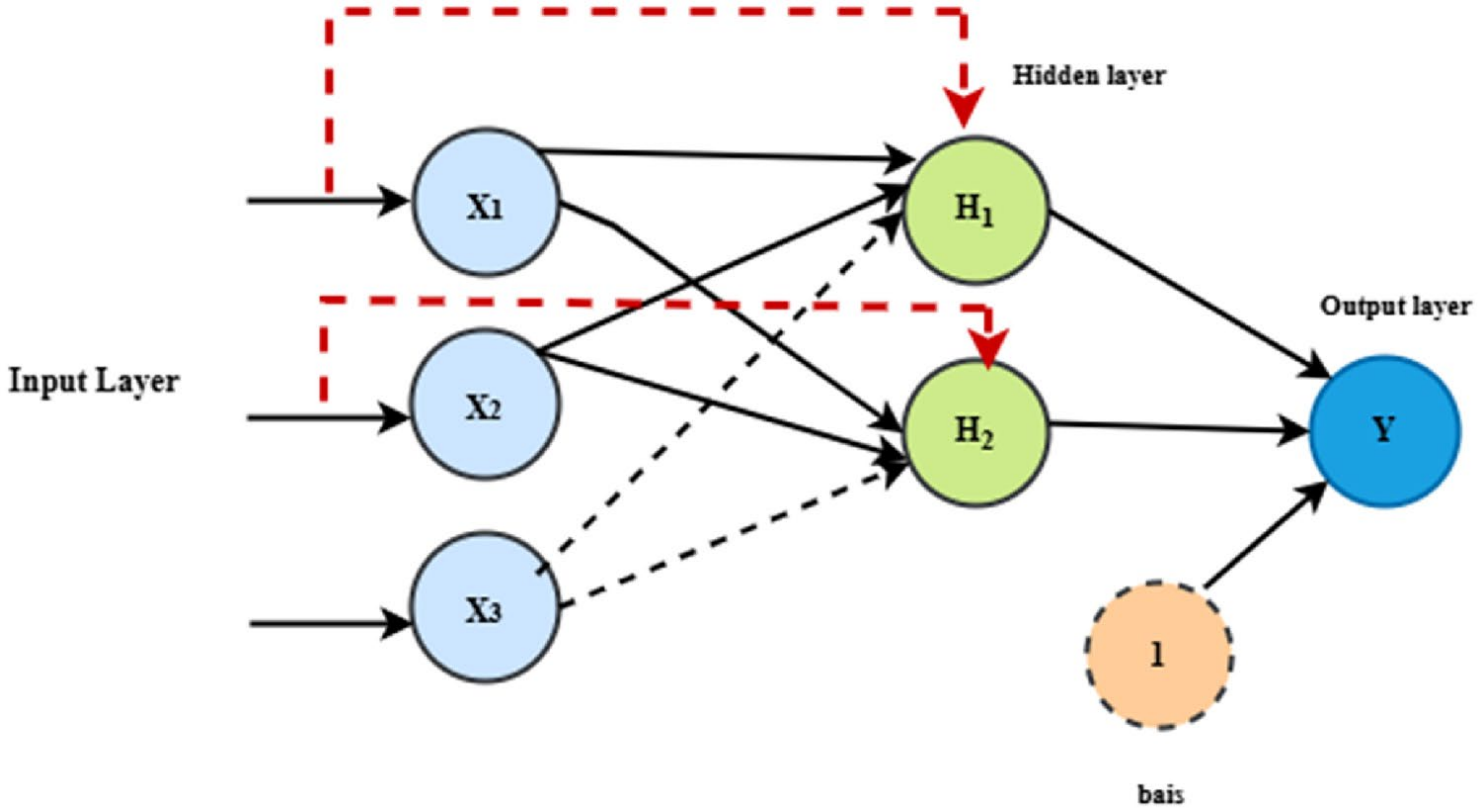
CFNN schematic diagram.

Although the architectures of CFNN and MLFFN are the same, a weight matrix joins each hidden layer in the network to the input signal ^46^. Figure 12 depicts the CFNN architecture. All hidden layers in the network, with the exception of the first hidden layer in CFNN, have two weight matrices that control the input and output signals from the upper layer of the NN, respectively ^47^.

The following is the formula for the equations developed from the CFNN model:

24 $${y}_{out}={\sum}_{i=0}^{i=n}{f}^{i}{w}_{i}^{i}{x}_{i}+{f}^{o}{\sum}_{j=1}^{k}{w}_{j}^{o}{f}_{j}^{h}({\sum}_{i=1}^{n}{w}_{ji}^{h}{x}_{i})$$

where ${w}_{i}^{i}$ is the weight from the input layer to the output layer, ${f}^{i}$ represents the activation function from the input layer to the output layer,${f}^{o}$ is the activation function of the output layer, and ${f}_{j}^{h}$ is an activation function of the hidden neurons. When a bias is applied to the input layer, and every neuron in the hidden layer has an activation function of ${f}^{h}$, Eq. (24) changes to the following formula ^31^.

25 $${y}_{out}={f}^{0}({w}^{b}{\sum}_{j=1}^{k}{w}_{j}^{o}{f}^{h}({w}_{j}^{b}+{\sum}_{i=1}^{n}{w}_{ji}^{h}{x}_{i})$$

The weights from the bias to the output and hidden layers are represented by ${w}^{b}$ and ${w}_{j}^{b}$, respectively.

**Fig. 13:**
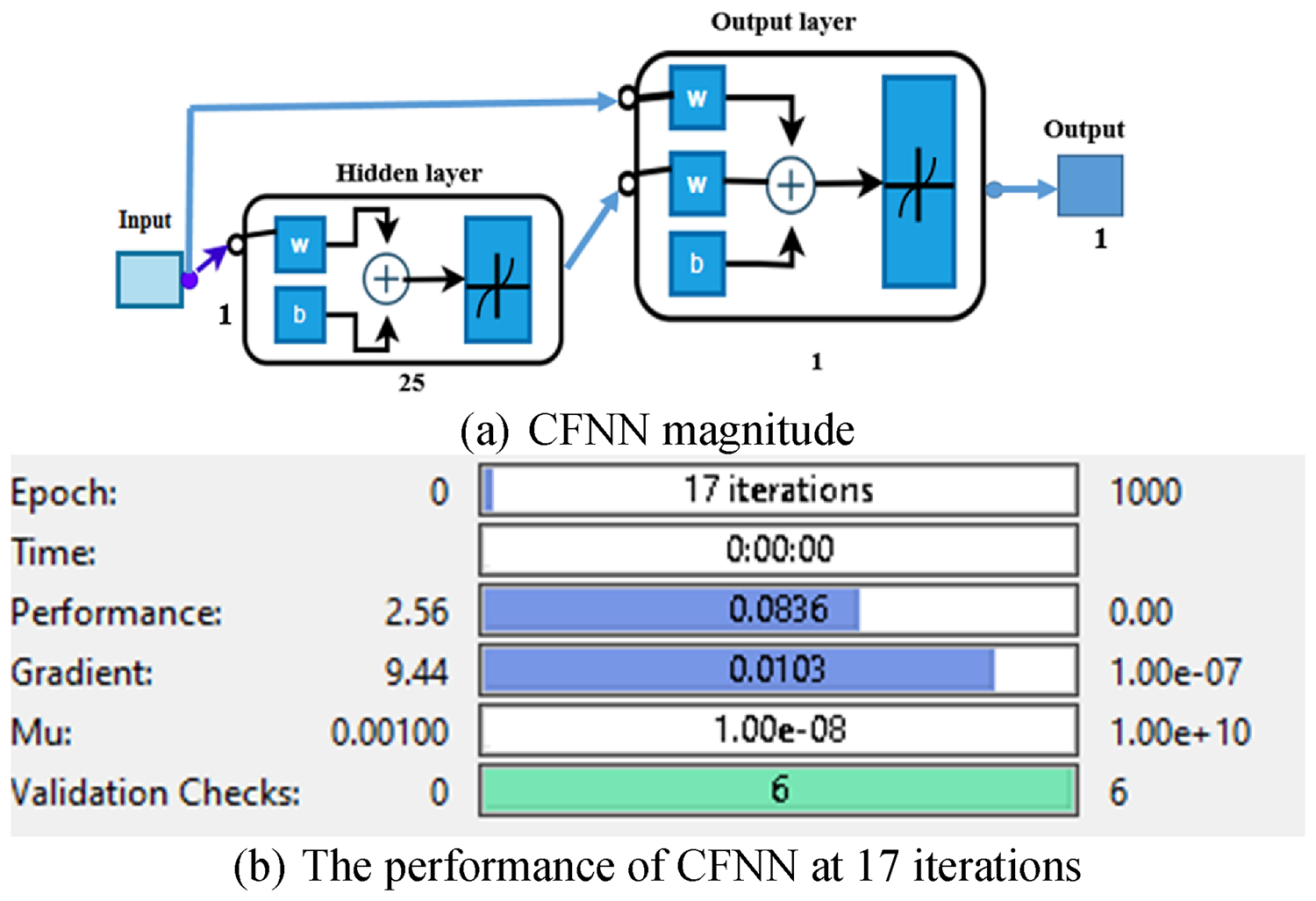
The NFTOOL parameters of CFNN.

**Fig. 14:**
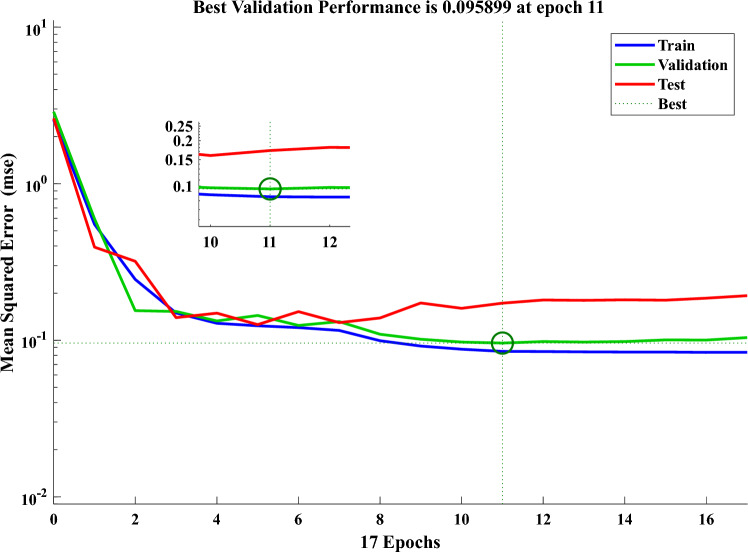
Integration of the studied system’s performance with CFNN.

After the CFNN has been established, the training process can be ended. To generate the desired output, the dataset is fed into the network. To commence the training process, the CFNN desired inputs, target results, weights, and biases, as stated in the architecture. The same characteristics that were used in MLFFNN are used in this type of NN. Figure 13 and Fig. 14 show the results of the training process of the CFNN. The error drops sharply during the initial epochs, showing rapid learning. The best validation performance is achieved around epoch 10, as indicated by the highlighted point. After this stage, the curves begin to stabilize with slight fluctuations. The close behaviour of training, validation, and test curves indicates good generalization with minimal overfitting.

#### Elman NN technique

**Fig. 15:**
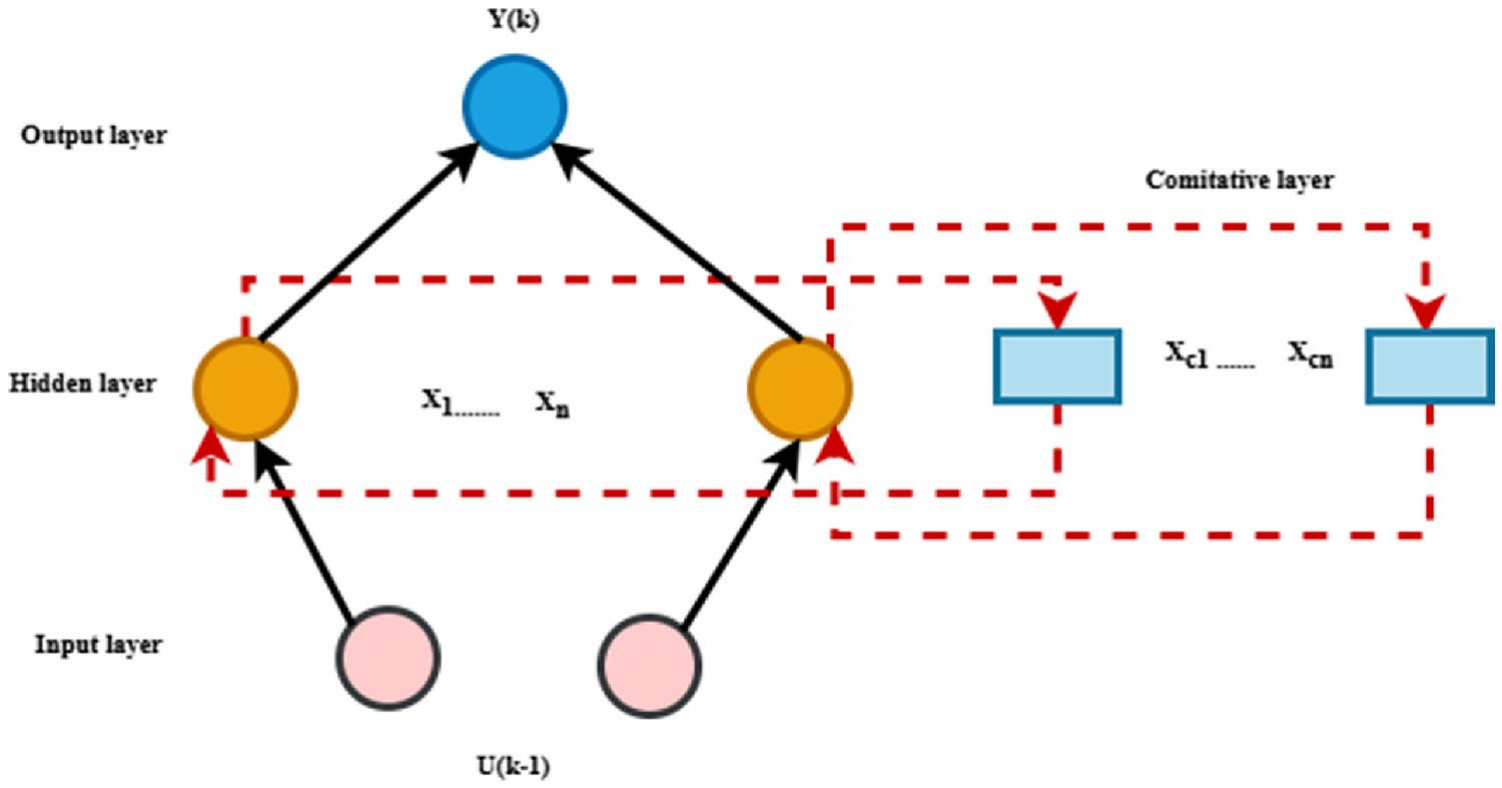
The architecture of the Elman NN.

Contextual data is collected and preserved in a hidden layer by the Elman Neural Network (Recurrent NN). A hidden layer, an output layer, and an input layer make up its three layers. Interfaces among the input and output layers and the hidden layer are established ^48,49^. By delivering the activation values to itself across several steps, the hidden layer keeps track of contextual data. The network can analyse patterns of data like time series or conversations because of its ability to retain information about preceding inputs over time, as shown in Fig. 15^50^. The output of hidden neuron j observed within the hidden layer of the NN is provided as ^31^:

26 $${y}_{j}={\Psi}_{j}\left({h}_{j}\right)={\Psi}_{j}({\sum}_{i=1}^{i=n}{w}_{ji}{x}_{i}+{\sum}_{j=1}^{j=n}{c}_{ji}{y}_{n}(k-1))$$

where ${x}_{i}$ is the input of the Elman NN,${w}_{ji}$ is the weight between the input and hidden neurons $j$ and ${c}_{ji}$ is the weight between the input ${y}_{n}(k-1)$ and hidden neurons j.

27 $${y}_{out}={\Psi}_{k}\left({\sum}_{j}^{n}{b}_{1j}{y}_{j}\right)=tanh\left({\sum}_{j}^{n}{b}_{1j}{y}_{j}\right)$$

where ${b}_{1j}$ is the weight between the hidden neurons and ${y}_{out}$ is the output power generated by the Elman NN.

**Fig. 16:**
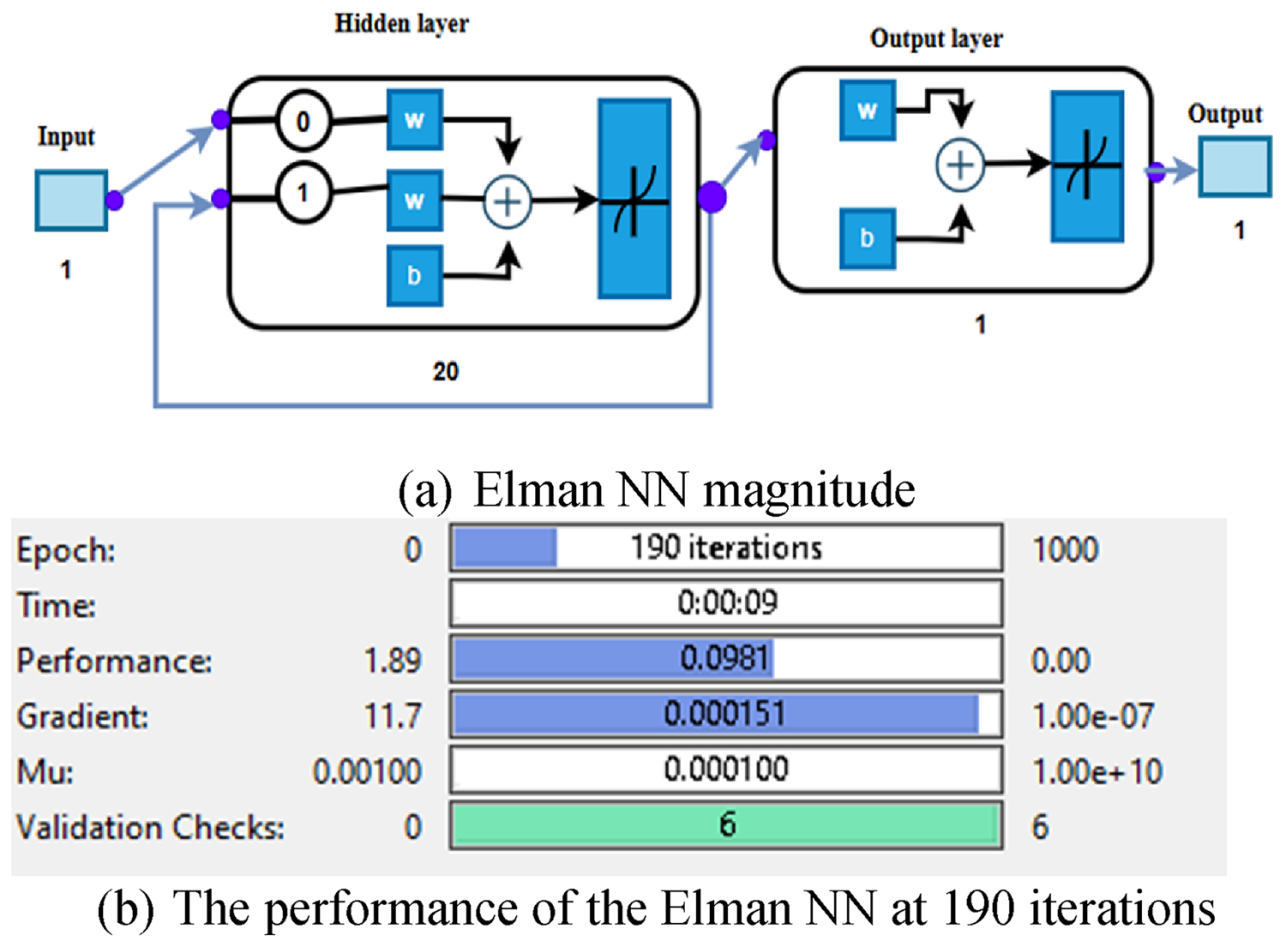
The NFTOOL parameters of Elman NN.

**Fig. 17:**
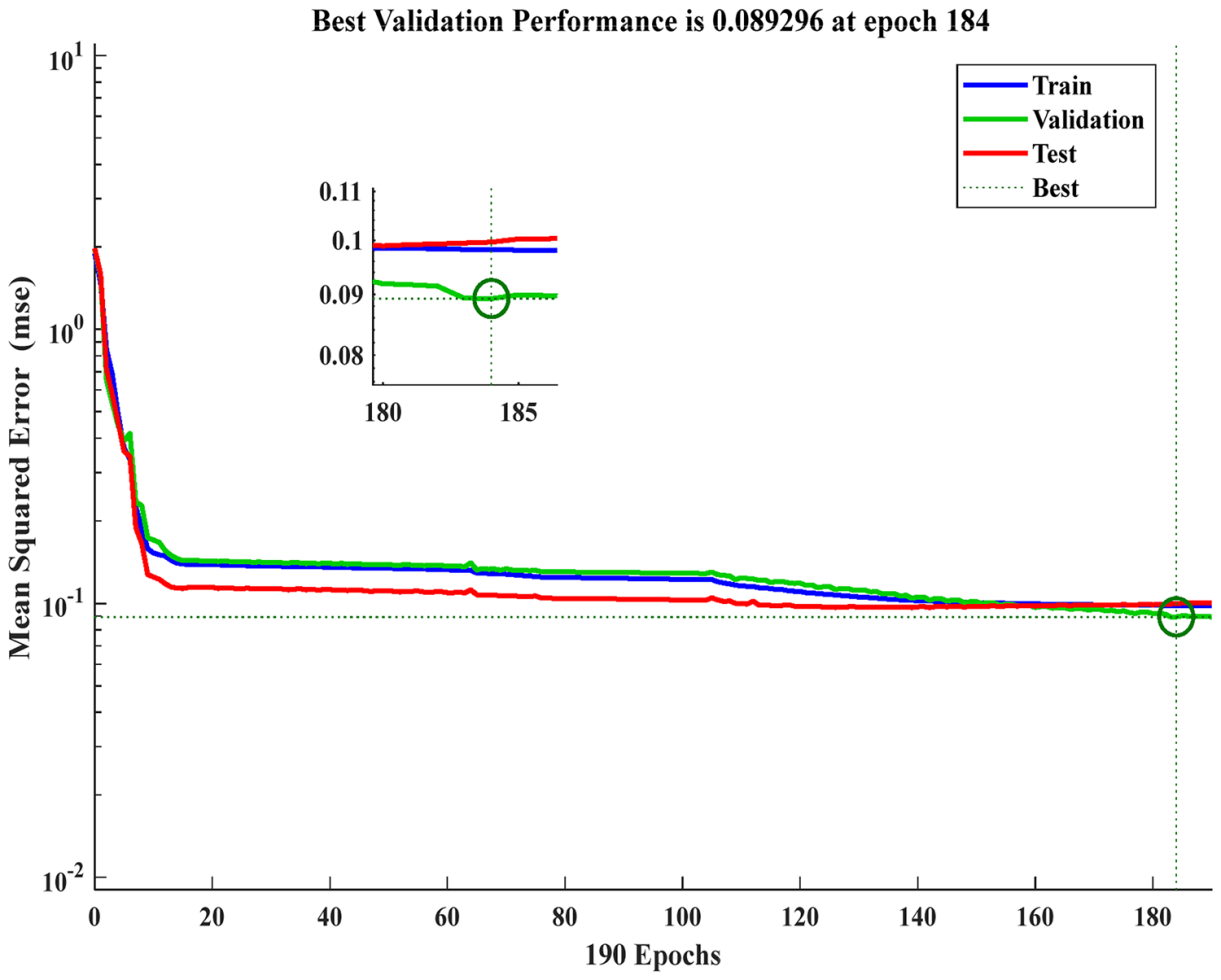
Integration of the studied system’s performance with the Elman NN.

The training process can be performed once the Elman NN has been established. The dataset is passed into the network to produce the desired result. As specified in the design, the Elman NN needs inputs, the expected result, and weights to initiate the training process. The outcomes of Elman’s training process are presented in Fig. 16 and Fig. 17. The error drops sharply at the beginning, reflecting fast learning, and then decreases until it reaches its minimum near the final epochs. The marked point indicates the best validation performance. The close overlap between the training, validation, and test curves demonstrates stable learning and strong generalization with no clear signs of overfitting.

Mean Square Error (MSE) was employed to evaluate the networks of the CFNN, Elman NN and MLFFNN models to judge the performance level of these approaches. The equation for calculating the MSE was expressed as follows^51,52^:

28 $$\mathrm{M}\mathrm{S}\mathrm{E} =\frac{1}{c}\sum_{i=1}^{c}({{y}_{desired}-{y}_{out})}^{2}$$

Where $c$ is the number of data points, ${y}_{desired}$ is the actual value and ${y}_{out}$ is the predicted value.

The MSE was calculated after the specified NNs were trained. The expected MLFFNN was trained, and the MSE was both lower and more effective than the other NN types. This meant that using the MLFFNN resulted in higher accuracy and approximation. However, the MSE of the CFNN and Elman NN architectures was additionally low and acceptable, as shown in Table 4.

**Table 4:** The optimum specifications for three constructed NNs.

Features	MLFFNN	CFNN	Elman NN
Hidden neurons	25	25	20
Iteration	9	11	184
MSE	0.0027224	0.095988	0.089296
Training method	LM algorithm	LM algorithm	LM algorithm

According to a result on Table 4, MLFFNN provides a blend of quick inference and modest training cost. The CFNN, considered a modification of MLFFNN, adds connections from each layer to every layer before it. With additional weights, this enhances the inference time and training cost. For the purpose of preserving temporal information, the Elman NN integrates feedback connections through backdrop units. Though featuring the highest computational cost of the three, this makes it suitable for time-series and sequential data types. As a result, Elman networks require a lot of computational power even though they are successful at dynamic tasks.

The model performance is influenced by key hyperparameters such as the number of hidden layers, neurons, and activation functions. These were selected empirically through preliminary experiments to balance accuracy and computational efficiency. The chosen architecture demonstrated stable performance, low mean squared error, and good generalization across wind speed profiles. However, a comprehensive sensitivity analysis was not conducted to quantify the impact of each hyperparameter. Future work will address this limitation through systematic analysis and the use of automated hyperparameter optimization techniques. The dataset was divided into training, validation, and testing sets. The consistency of results across these subsets, along with the close agreement between training and validation errors, indicates good generalization. Additionally, model complexity was carefully controlled. Although advanced regularization methods such as dropout were not applied, the model achieved stable and reliable performance.

There are some limitations of using NN techniques, including:

- Significant amounts of quality training data are required for neural networks to work efficiently.
- They frequently make decisions that are difficult to comprehend.
- It can be highly computational to train and carry out large neural networks.
- Using NNs to guarantee control system stability under dynamic circumstances is not straightforward.
- Outside of the training input space, they might demonstrate unpredictable behaviour.

**Fig. 18:**
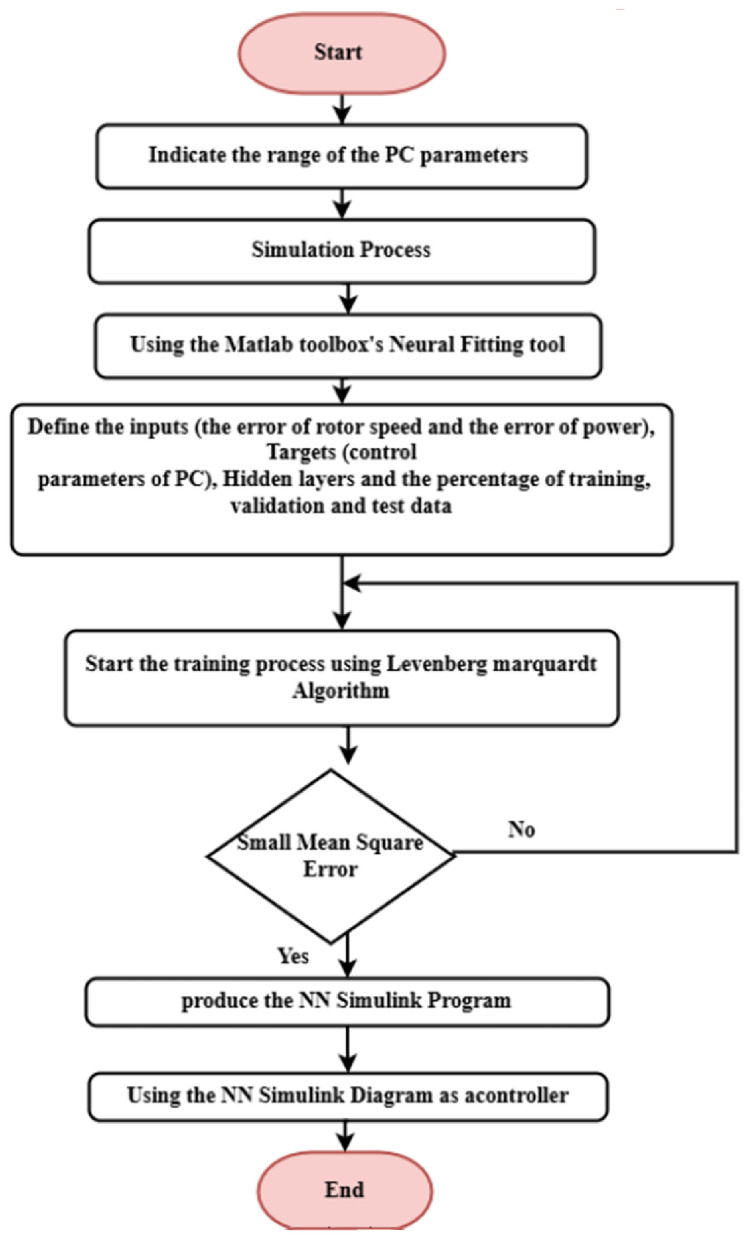
The flowchart for applying NN to customise the PC’s parameters.

Based on the previous study of NN types, MATLAB Neural Network Toolbox in conjunction with MLFFNN, CFNN and Elman NN is employed to serve as a wind turbine system controller. Figure 18 shows the flowchart for applying ANNs to customize the PC’s parameters.

**Fig. 19:**
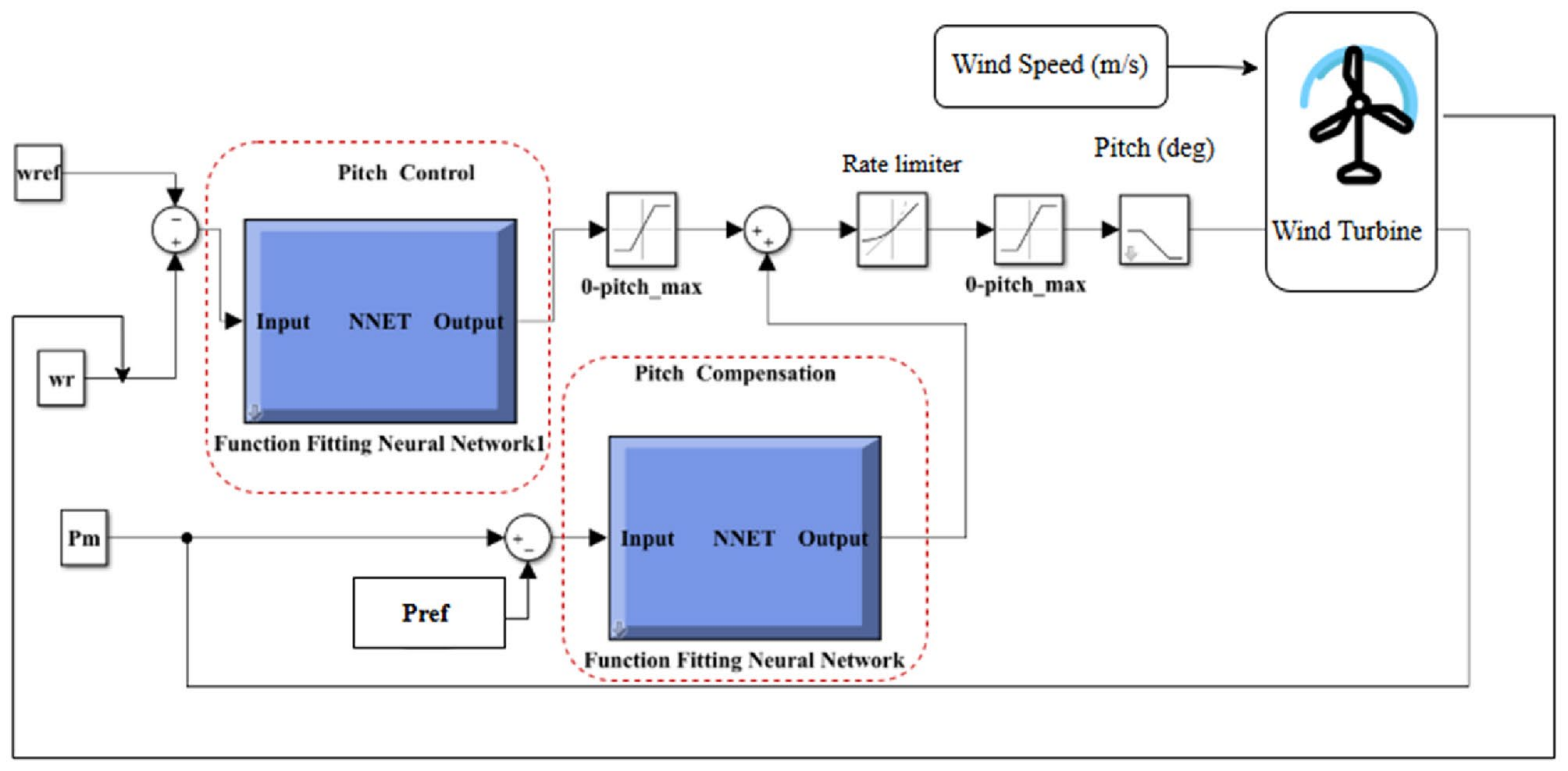
The pitch angle NN controller.

As shown in Fig. 19, MLFFNN, CFNN, and Elman controllers were used to implement the pitch angle in the wind turbine system using MATLAB SIMULINK.

## Simulation results and discussions

This section shows the results from two different stages: The NN models were trained and tested with real wind speed as the initial step. Three cases of wind speed variation are applied to a MATLAB/SIMULINK model of the WT system, and the results of each case after applying different types of control are clarified in the second stage.

### NN schemes performance study

**Fig. 20:**
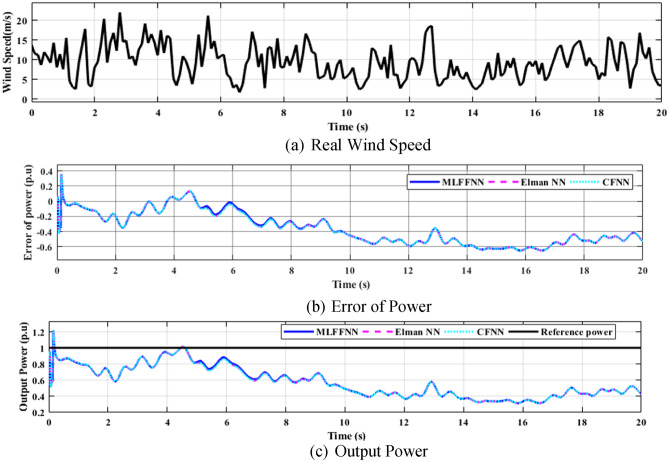
Wind turbine efficiency based on ANNs MLFFNN, Elman NN and CFNN.

The three neural network types (MLFFNN, CFNN and Elman NN) were assessed with actual wind speed^53^ that was applied in MATLAB wind turbine system with rated wind speed $12 m/s$ , air density 1.225 kg/m^3^ and rated power $1 p.u$, as seen in Fig. 20. The output power results indicate that all three controllers exhibit high performance; however, the MLFFNN achieves the superior results, as summarised in Table 5.

**Table 5:** Output power error at its maximum, minimum, mean, and standard deviation values based on MLFFNN, CFNN and Elman NN.

Parameters	MLFFNN	Elman NN	CFNN
Mean error	-0.3572	-0.3594	-0.3596
Maximum error	0.3584	0.3587	0.3585
Minimum error	-0.6521	-0.6526	-0.6522
Standard deviation	0.2132	0.2133	0.2143

### Comparative Study of Different Control Strategies

For computational simulation, PC systems apply the Simulink component, which operates on MATLAB Software. The values that follow are the WT’s parameters; the system includes six wind turbines with three blades each; the rated wind speed is $12 m/s$. The rated output power is $1.5 MW(1 p.u)$, frequency of the grid is $60 HZ$, ideal TSR is $8.1$. The perfect power coefficient is $0.48$, and maximum pitch angle $27^\circ$ . We have implemented a step, ramp, and random wind speed in the WT system.

#### Step profile variations

**Fig. 21:**
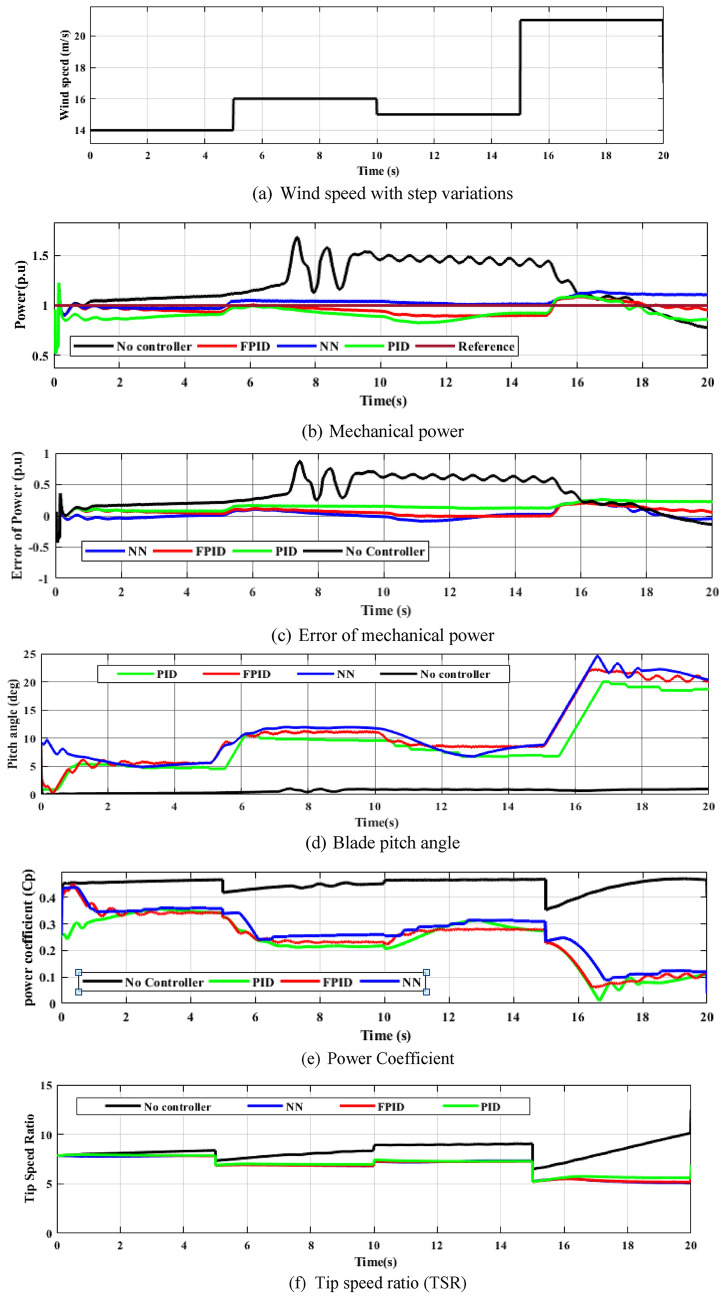
Wind turbine efficiency during step variation.

Figure 21 shows the step variations in wind speed, pitch angle changes due to wind speed variations, power coefficient, TSR, mechanical power, and the DFIG’s mechanical power error. Pitch-angle control strategies perform successfully based on wind turbine characteristics. Pitch-angle is close to zero degrees $\left(\beta =0^\circ \right).$ When wind speed is between cut-in and rated values, which causes the TSR and the power coefficient in its perfect values of 8.1 and 0.48, respectively.

**Fig. 22:**
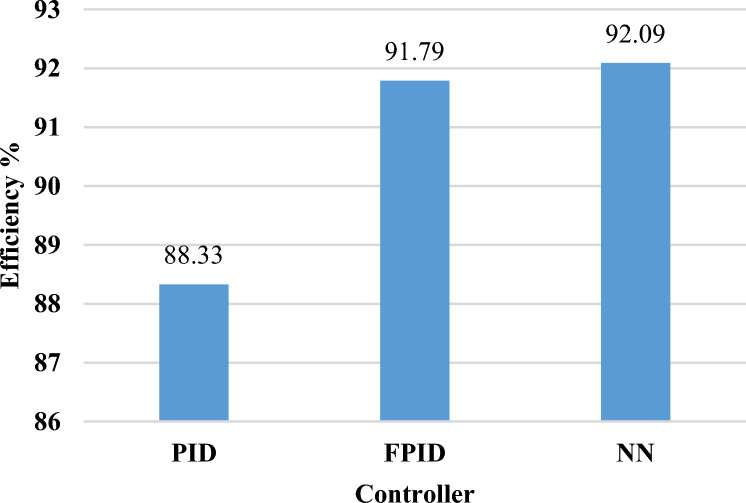
The efficiency of the WT system based on output mechanical power for a step variation.

Consequently, as wind speed alters, the mechanical output power achieves its maximum level. The controllers actively modify the pitch angle to reduce the power coefficient when the wind speed crosses the rated value to preserve the output mechanical power at its optimum level (1 P.U = 1.5 MW) and prevent the WT opposed to fatigue damage. As the efficiency of WT to achieve the rated value of output power ($1 p.u$) is satisfactory and equal to 98.9% when the NN controller was applied with a step variation of Wind speed, while the FPID efficiency is 93.24% and the PID efficiency is 91.84%, as displayed in Fig. 22 and Table 6, which was calculated by the following equation^54^.

29 $$\eta = \frac{{P_{m} }}{{P_{rated} }}\%$$

where ${P}_{rated}$ is the rated power and ${P}_{m}$ is the measured power.

**Table 6:** The efficiency of the WT system based on output mechanical power.

Control strategy	Output mechanical power (${\boldsymbol{p}}.{\boldsymbol{u}}$)	Efficiency (%)
PID	0.9184	91.84
FPID	0.9324	93.24
NN	0.9890	98.9

The system performance with NN in Fig. 21(b) is often faster compared to that of the PID and FPID Controllers, with a settling time of 2.3 s and steady state error of 0.2% as presented in Table 7. So NN is the best controller compared to PID and FPID. Table 8 displays the mean, maximum, minimum and standard deviation of the absolute error of each strategy according to Fig. 21 (c). The MAE and Std. were calculated by the following equations ^49^:

**Table 7:** Analysing the differences between the NN, PID and FPID approaches for WECS.

Controller	Settling time (seconds)	Steady state error (%)
PID	4	10
FPID	3	0.5
NN	2.3	0.2

**Table 8:** The WECS output power value at its maximum, minimum, mean, and standard deviation.

Parameters	NN	FPID	PID
Mean error	0.0111	0.0676	0.0816
Maximum error	0.3615	0.3588	0.3628
Minimum error	-0.4271	-0.4295	-0.4295
Standard deviation	0.0966	0.0646	0.0665

30 $$MAE=\frac{1}{c}\sum_{i=1}^{c}\left|{y}_{desired }-\left.{y}_{out}\right|\right.$$

31 $$Std.=\sqrt{{\sum}_{i=1}^{c}\frac{{({y}_{desired}-MAE)}^{2}}{c-1}}$$

The NN controller achieves the lowest absolute error compared to both PID and FPID controllers, indicating superior accuracy and control performance. This improvement is mainly due to its ability to learn nonlinear relationships and adapt to changing operating conditions in real time.

#### Ramp profile variations

**Fig. 23:**
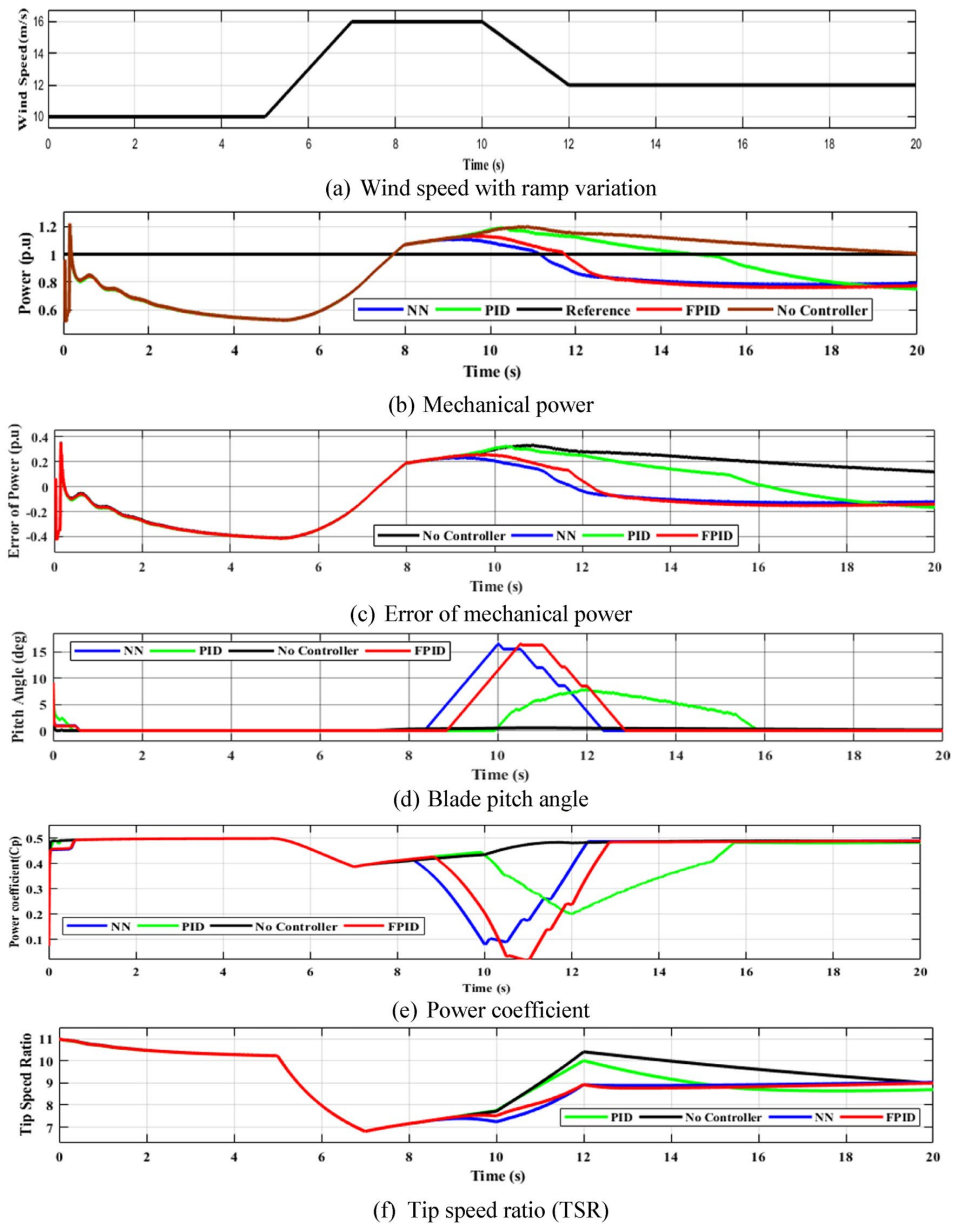
Wind turbine efficiency during ramp variation.

The Ramp wind-speed fluctuation pattern was employed to study the efficiency of control procedures that were put into operation. The performance of the turbine over the ramp fluctuation in wind speed is shown in Fig. 23. The mechanical power of the wind turbine is controlled to preserve the desired level. As seen in Fig. 23 (b), all targets of the pitch-angle control approaches were met because the output mechanical power remains approximately the same at ($$1 p.u)$$ at a larger rated wind speed (12 m/s).

**Fig. 24:**
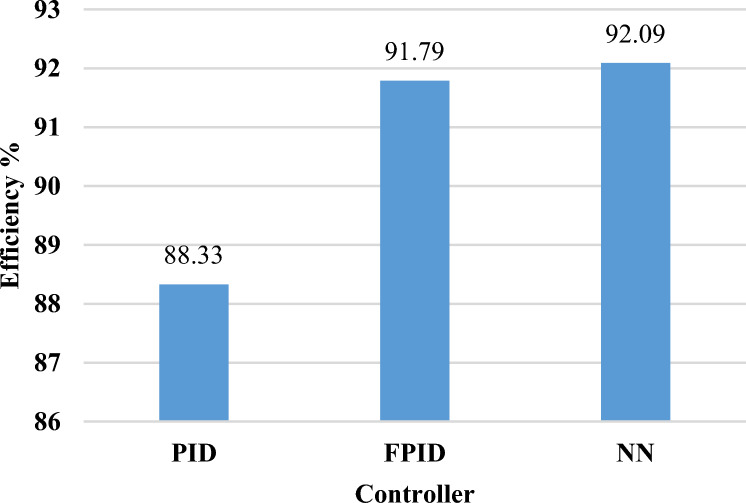
The efficiency of the WT system based on output mechanical power for ramp variation.

When the wind speed exceeds $12 m/s$ the output mechanical power was continually regulated to be developed at its maximum design value by decreasing the power factor. The efficiency of the output power of the NN strategy (92.02%) is larger than FPID (91.79%) and PID (88.33%), as presented in Fig. 24 and Table 9. The mean, maximum, minimum and standard deviation absolute error of each strategy according to Fig. 23 (c) are shown in Table 10. When evaluating the NN controller against PID and FPID, it delivers the lowest absolute error.

**Table 9:** The efficiency of the WT system based on output mechanical power for ramp variation.

Control Strategy	Output Mechanical power (${\boldsymbol{p}}.{\boldsymbol{u}}$)	Efficiency (%)
PID	0.8833	88.33
FPID	0.9179	91.79
NN	0.9209	92.09

**Table 10:** The maximum, minimum, mean, and standard deviation of the WECS output power value for ramp variation.

Parameters	PID	FPID	NN
Mean error	0.1167	0.0821	0.0791
Maximum error	0.3541	0.3612	0.3528
Minimum error	-0.4134	-0.4206	-0.4231
Standard deviation	0.1768	0.1753	0.1932

The improvement of the proposed MLFFNN controller over the FPID controller under ramp wind-speed conditions is limited due to the smooth and predictable nature of ramp inputs, where conventional controllers already perform well. However, the strength of the MLFFNN controller lies in its consistent performance across varying conditions. It shows more significant improvements under highly dynamic wind profiles, such as random variations, where adaptability is essential. Therefore, despite marginal gains in ramp conditions, the MLFFNN controller offers superior robustness and adaptability in realistic, fluctuating environments.

#### Random profile variations

**Fig. 25:**
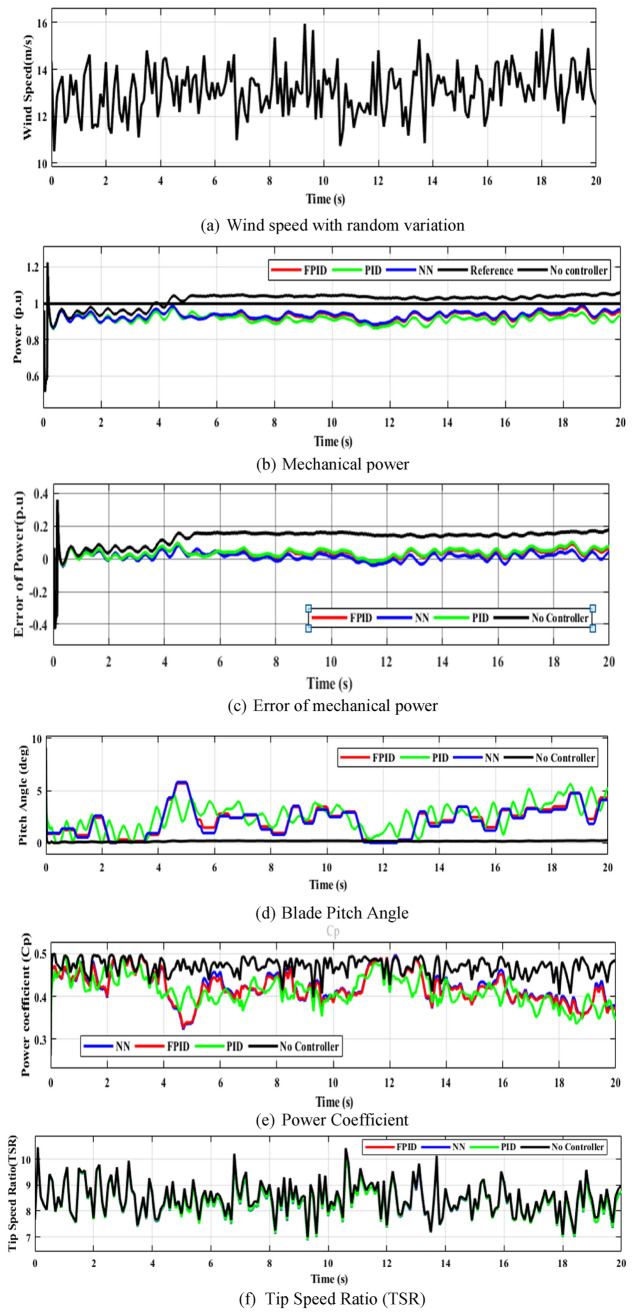
Wind turbine efficiency during random variation.

The efficiency of implemented restrictions was examined using the Random wind-speed fluctuation pattern. Figure 25 demonstrates the turbine’s performance over the wind speed random variation. The wind turbine’s mechanical power is monitored to keep it at the appropriate level. As can be shown in Fig. 25(b), at larger rated wind speeds (12 m/s), the output mechanical power remains nearly constant at $1 p.u.,$ indicating that all aims of the pitch-angle control strategies were achieved.

**Fig. 26:**
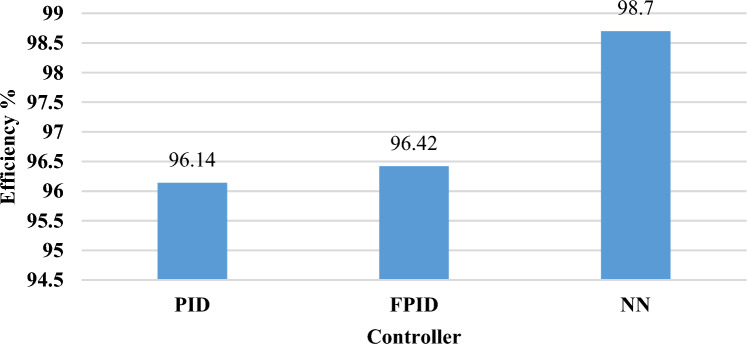
The efficiency of the WT system based on output mechanical power for random variation.

When the wind speed exceeds 12 m/s, the output mechanical power is periodically controlled to be generated at its highest design value by dropping the power factor. The efficiency of the output power of the NN technique (98.7) is larger than FPID (96.42) and PID (96.14), as indicated in Fig. 26 and Table 11. According to Fig. 24(c), Table 12 represents the mean, maximum, minimum, and standard deviation of the absolute error of each approach. When compared with PID and FPID controllers, the NN controller produces the lowest absolute error, indicating higher accuracy and better control performance. This is due to its ability to capture system nonlinearities and adapt to changing conditions more effectively than conventional controllers.

**Table 11:** The efficiency of the WT system based on output mechanical power for random variation.

Control strategy	Output mechanical power (${\boldsymbol{p}}.{\boldsymbol{u}}$)	Efficiency (%)
PID	0.9614	96.14
FPID	0.9642	96.42
NN	0.987	98.7

**Table 12:** The maximum, minimum, mean, and standard deviation of the WECS output power value for random variation.

Parameters	PID	FPID	NN
Mean error	0.0386	0.0358	0.013
Maximum error	0.3609	0.3590	0.3587
Minimum error	-0.4305	-0.4295	-0.4305
Standard deviation	0.0386	0.0368	0.0384

## Comparison with Previous Research

The comparison of the research clearly indicates that the multilayer MLFFNN used in this study behaves substantially better than conventional and previously suggested control systems for wind turbine pitch-angle control. To evaluate the controller behaviour under different operating conditions, rather than depending just on a single wind scenario, the comparison is based on the controller performance under multiple wind speed profiles (step, ramp, and random). This makes it possible to evaluate the proposed controller approach’s efficiency and resilience in greater detail. MLFFNN displays its effectiveness and stability with an efficiency of approximately 98.9% under step wind speed profiles and excellent performance across ramp and random profiles. Also, the quite low MSE (0.0027) and confirm the learning ability. In contrast with previous studies by Sitharthan et al. ^55^, Ali et al. ^56^, Ahmed A. Lasheen and Mahmoud M. Elnaggar ^57^ and Bekiroglu, E., et al. ^58^, Ahmed A. Lasheen and Mahmoud M. Elnaggar ^57^ achieved high efficiency when tested with a random wind speed profile, unlike the current study, which evaluates multiple speed profiles. The present neural network-based technique demonstrates enhanced reliability, adaptability, learning ability and dynamic accessibility, establishing it as the most potential choice among all the studied approaches, as shown in Table 13. The assessment does not directly provide a numerical benchmark; instead, it indicates performance trends.

**Table 13:** Comparison with previous studies.

Paper	Year	Control Technique	Speed profile	Efficiency (%)	Best performance approach	MSE	Learning ability
Current study	2025	PID	Step	91.84	MLFFNN	0.0027224	Yes
			Ramp	88.33			
			random	96.14			
		FPID	Step	93.24			
			Ramp	91.79			
			random	96.42			
		MLFFNN	Step	98.9			
			Ramp	92.09			
			Random	98.7			
Sitharthan, R., et al. ^55^	2017	PI	Random	Not reported	FFBP	1.54	Yes
		Fuzzy	Random				
		FFBP	Random				
Ali, Mustafa M., et al. ^56^	2020	PI	Step	84	MRC	Not reported	No
		MRC	Step	87			
Ahmed A. Lasheen and Mahmoud M. Elnaggar ^57^	2020	Traditional PI	Random	99.96	MPC	Not reported	No
		MPC	Random	99.987			
Bekiroglu, E., et al. ^58^	2022	PI	Random with rated speed 12.9 m/s	94	PI + MPPT controller	Not reported	No
		PI + MPPT controller	Random with rated speed 12.9 m/s	100			

## Conclusions and Future Work

This study evaluated the dynamic stability of pitch-angle control in a DFIG-based wind turbine using PID, fractional PID (FPID), and NN controllers. Three different types of NNs (MLFFNN, CFNN, and Elman NN) were implemented and tested using real wind speed data. Simulation results clearly demonstrate that all three NN types of significantly improved control system performance compared to traditional methods, with the MLFFNN outperforming the other approaches. The MLFFNN achieved the lowest mean square error of 0.0027024, indicating higher accuracy and control precision. Further analysis revealed that pitch-angle strategies using NN-based controllers effectively preserved mechanical power and reduced aerodynamic loads. Among the evaluated methods, MLFFNN proved to be the most suitable for achieving these objectives. It enabled smoother pitch control, reduced power peaks, and minimized fatigue loads, which are critical for the long-term reliability of wind turbines. Under step changes in wind speed, the MLFFNN achieved the highest efficiency in maintaining output power at the rated value of 1 p.u. Similarly, when subjected to ramp and random wind speed variations, the MLFFNN again delivered superior performance.

The studied results confirm that the NN-based control strategy, especially MLFFNN, provide the best dynamic adaptability and accuracy. Moreover, the NN controller successfully reduced mechanical stress on the wind turbine during both ramp and step wind speed variations by adaptively adjusting the pitch angle in response to fluctuations in the power coefficient. This adaptive behaviour not only enhances energy capture but also safeguards the structural integrity of the turbine. In conclusion, the assessment justifies the superiority of the MLFFNN-based control system over FPID and PID strategies. It offers the highest control precision, improved power efficiency, and the best mechanical protection, making it a highly effective solution for variable-pitch control in modern wind turbines. Finally, Experimental validation and real-time implementation with real-world factors such as sensor noise, actuator saturation and delay, communication latency, and mechanical wear are planned for future work.

## Data Availability

By request, the authors will provide the raw data used to demonstrate the outcomes of this paper.

## Abbreviations

DFIG: Doubly-fed induction generator
WECS: Wind energy conversion system
PC: Pitch control
WT: Wind turbine
PID: Proportional integral derivative controller
FPID: Fractional PID
ANN: Artificial neural network
MLFFNN: Multilayer feedforward neural network
CFNN: Cascade forward neural network
TESs: Traditional energy sources
RESs: Renewable energy sources
BP-PID: Back propagation PID
FSWT: Fixed speed and variable speed
VSWT: Variable speed wind turbine
PMSG: Permanent magnet synchronous generators
FFBP: Feed-forward back propagation
MRC: Model reference control
MPC: Model predictive control
MSE: Mean square error
MAE: Mean absolute error
Std.: Standard deviation
